# Blood feeding, microbiota, and layered mosquito immunity: from pathway phenotypes to vector competence

**DOI:** 10.3389/fimmu.2026.1885857

**Published:** 2026-07-27

**Authors:** George-Rafael Samantsidis, Nicolas Buchon

**Affiliations:** Department of Entomology, Comstock Hall, Cornell University, Ithaca, NY, United States

**Keywords:** *Aedes aegypti*, *Anopheles gambiae*, anticipatory immunity, blood feeding, microbiota, mosquito immunity, RNA interference, vector competence

## Abstract

Mosquitoes impose a significant epidemiological burden on public health through the transmission of malaria parasites and arboviruses. Vector competence, the ability of a mosquito to acquire, maintain, and transmit pathogens, is tightly linked to immune regulation, but immune phenotypes in mosquitoes cannot be interpreted only as pathogen-triggered activation of canonical pathways. Following blood feeding, female mosquitoes undergo extensive physiological remodeling, including endocrine reprogramming, microbiota shifts, oxidative and barrier stress, digestive and metabolic activation, and coordinated modulation of immune function. Because pathogen acquisition occurs within this same physiological window, mosquito immunity must be interpreted in relation to the blood-fed state in which infection unfolds. In this review, we provide a conceptual synthesis of mosquito immune function around this blood-meal-centered framework, with emphasis on how microbiota dynamics, epithelial physiology, hemocyte activity, and systemic endocrine and nutritional signals shape immune capacity and vector competence. We then examine how conserved immune modules, including Toll, IMD, JAK/STAT, JNK, RNA interference, and complement-like systems, act against bacteria, fungi, malaria parasites, and arboviruses, highlighting the distinction between pathway activation, effector function, tissue tolerance and repair, altered permissiveness, and pathogen restriction. We further discuss juvenile hormone, ecdysone, insulin-like signaling, and vertebrate-derived blood factors as systemic inputs that shape immune competence rather than reproduction alone. Finally, we propose that blood feeding can establish an anticipatory immune state, defined here as physiological preconditioning by recurrent feeding-associated cues rather than pathogen-specific prediction. This framework helps explain why the same immune pathways can restrict infection, preserve tissue integrity, or indirectly modulate transmission by altering tissue permissiveness.

## 1 Introduction

### 1.1 Vector immunity is built around the blood meal

Mosquitoes are responsible for a disproportionate burden of human infectious diseases due to the wide range of pathogens transmitted during blood-feeding. These include viruses such as yellow fever, Zika, dengue, and chikungunya, transmitted primarily by mosquitoes of the genus *Aedes*, as well as protozoan parasites such as *Plasmodium* spp., the causative agents of malaria that are transmitted by certain species of anopheline mosquitoes. Together, vector-borne diseases account for a substantial fraction of the global infectious disease burden, estimated at approximately 17% of all infectious diseases (1), with mosquitoes representing some of the most important vectors. Although vector control strategies have substantially reduced transmission in multiple regions, the emergence of insecticide resistance in mosquito populations (2, 3) and drug resistance in *Plasmodium* parasites (4) have contributed to renewed transmission and control failure. In parallel, arboviral diseases have expanded geographically, driven in large part by vector range expansion, urbanization, and environmental change, which increase opportunities for transmission (5–10).

These epidemiological realities have placed vector competence, the ability of a mosquito to acquire, maintain, and transmit a pathogen, at the center of infection biology. Yet vector competence is not a fixed property of the mosquito or the pathogen alone. It emerges from interactions among mosquito genotype, pathogen genotype, microbiota (11–13), environmental history, nutrition, reproductive state (14, 15), and tissue physiology (16–18). For immunology, this creates a central interpretive challenge: immune phenotypes measured in infected mosquitoes may reflect direct pathogen recognition, but they may also be shaped by the physiological consequences of blood feeding, including microbiota expansion, endocrine signaling, and tissue damage and repair processes.

The dominant framework for insect immunity has largely been established in *Drosophila*, where microbial recognition activates conserved signaling pathways such as Toll, Immune Deficiency (IMD), Janus kinase/signal transducer and activator of transcription (JAK/STAT), and c-Jun N-terminal kinase (JNK), which regulate antimicrobial peptide production, cellular immunity, epithelial renewal, and stress responses (19–23). This framework has provided essential conceptual and experimental language for insect immunology and has been productively translated across species. However, mosquitoes require additional interpretation because hematophagy places immune signaling within a distinct physiological context. Female mosquitoes alternate between sugar feeding and blood feeding, and the blood meal itself constitutes a large-scale transition involving nutritional overload, mechanical distension, oxidative stress, microbial expansion, endocrine activation, and reproductive reprogramming (24–26). Importantly, pathogen acquisition occurs within the same temporal window as this transition. As a result, conserved immune pathways in mosquitoes are not always deployed against infection in isolation, but within a blood-feeding context that can reshape the canonical insect-immunity models.

This difference matters because, in mosquitoes, blood feeding is itself an immune-relevant physiological event. A blood meal drives expansion and restructuring of the resident microbiota (27–29), induces formation of the peritrophic matrix, alters midgut redox homeostasis, activates digestive and detoxification programs (30, 31), and triggers extensive remodeling of epithelial and basal lamina architecture (32–34). In parallel, it engages endocrine pathways such as insulin–insulin-like growth factor signaling (IIS) and ecdysone (20E) signaling, classically associated with vitellogenesis and egg development (35–37), while also reshaping hemocyte activity (38–43) and circulating immune effector production (44–46). During this same physiological window, mosquitoes acquire infectious blood meals containing viruses, such as arboviruses, or parasites, such as *Plasmodium* spp., placing pathogen exposure within a dynamically reprogrammed microbial, endocrine, epithelial, and immune context. Although pathogen exposure, particularly during *Plasmodium* infection, can elicit immune responses that differ from those induced by blood feeding alone (47–49), this does not imply a simple separation between “blood-meal immunity” and “pathogen immunity.” Instead, immune outputs arise from the integration of multiple concurrent inputs, including pathogen-derived signals, microbiota dynamics, blood-meal-induced physiological changes, and tissue stress responses. Accordingly, vector competence should not be reduced to pathogen-triggered immunity alone, but instead emerges as a composite phenotype shaped by layered signals that influence resistance, tolerance, repair, tissue permissiveness, and transmission.

Building on this framework, mosquito immunity can be interpreted as a physiological system whose functional output is shaped by an integrated blood-meal–induced state encompassing metabolic, endocrine, microbiota, and tissue-level conditions. In this usage, building on prior descriptions of blood feeding as an anticipatory defense response (45), “anticipatory immunity” does not imply pathogen-specific prediction, but rather physiological preconditioning by recurrent signals associated with blood-feeding. In this framework, conserved signaling pathways remain central, but their functional roles are state-dependent, varying with the inducing signals, tissue, timing, and stage of infection. Rather than functioning as isolated signaling modules, immune pathways must therefore be understood in terms of what they do, where and when they are active, which cues induce them, and how they ultimately shape pathogen transmission.

The relevance of mosquito immune pathways is directly reflected in vector competence, as manipulation of specific immune modules can alter infection dynamics and reduce pathogen transmission. This principle has motivated a range of biological control strategies, including gene-drive technologies and microbial-based strategies, such as *Wolbachia* deployment. However, the outcomes of such interventions are often context-dependent, with variable efficacy observed across ecological settings and transmission systems, including heterogeneous performance in highly endemic regions (50). Such variability is consistent with a broader feature of mosquito-pathogen interactions, in which vector competence is not a stable trait that can be inferred solely from genotype or single-pathway perturbations, but a phenotype that is highly sensitive to physiological state, feeding history, microbiota composition, and environmental conditions. As a result, experimental measures of vector competence can differ across studies even when genetic backgrounds and pathogens are comparable, reflecting differences in underlying mosquito physiological and microbial states. This perspective helps explain why pathway-centric interpretations of immune perturbation often fail to predict transmission outcomes robustly across contexts. It further suggests that some inconsistencies in vector competence assays and intervention trials may arise not from experimental noise alone, but from unaccounted variation in mosquito physiological state at the time of infection or measurement. Therefore, recognizing vector competence as a state-dependent immune phenotype enables a more mechanistically informed approach to both experimental design and intervention development, by aligning strategies with the layered immunological, physiological, and ecological determinants of pathogen transmission.

To further develop this perspective, we have organized this conceptual review in four steps in order to provide representative examples that support a blood-meal centered framework of mosquito immune system and vector competence. First, we establish why immune phenotypes in mosquitoes cannot be read directly as pathway-mediated defense. Second, we examine how the blood-fed midgut and resident microbiota create a microbial, redox, epithelial, and tissue-remodeled landscape in which pathogens encounter distinct barriers. Third, we reassess conserved pathways and effector modules under tissue, cue, and pathogen-stage specificity. Finally, we integrate endocrine regulation, epithelial repair, hemocyte biology, and experimental priorities into a model of state-dependent immunity and vector competence. Across these sections, we emphasize that genes and pathways may be required for vector competence without functioning as direct pathogen sensors or antipathogen effectors. Although this framework is intended to apply broadly to hematophagous mosquitoes, much of the mechanistic evidence discussed here comes from *Aedes aegypti* and *Anopheles gambiae*, with selected support from other vector systems. A central practical implication is that studies of mosquito vector competence should distinguish the inducing cue, pathway activation, effector output, tissue state, pathogen stage, and transmission-relevant outcome rather than treating them as a single immune phenotype.

### 1.2 The importance of distinguishing pathway phenotypes from pathway-mediated defense

A recurrent challenge in mosquito immunity is that similar experimental outcomes can support multiple causal interpretations. Genetic perturbations or correlations between pathway activity and pathogen load can demonstrate that a factor influences infection outcome, but they do not always establish whether the pathway is directly activated by the pathogen, nor whether the resulting phenotype reflects resistance, tolerance, tissue repair, or altered permissiveness. This ambiguity complicates the mechanistic attribution of vector-competence-related phenotypes, since the same outcome can arise from distinct underlying physiological processes. As a result, linking observed immune responses to specific biological mechanisms requires careful consideration of the physiological and microbial context in which these phenotypes emerge.

In the sugar-fed adult stage, the mosquito is not immunologically quiescent. The midgut harbors resident microbial communities whose composition varies with species, environment, diet, and developmental history. These communities are associated with multiple phenotypic outcomes, including larval development, adult physiology, fecundity, longevity, and vector competence (11, 51–56). In addition to these resident communities, mosquitoes continuously acquire environmental microbes during sugar feeding, which can transiently alter microbiota composition and, in some cases, promote dysbiosis. Even before a blood meal, microbial products and metabolites can shape epithelial homeostasis, immune gene expression, and systemic physiology. As a result, infection phenotypes are not measured against a neutral baseline, but against a preconditioned and dynamically modulated physiological and microbial state.

This complexity is further reinforced at the level of immune gene architecture. Comparative genomics has revealed substantial expansion and diversification of immunity-related gene families in mosquitoes, including thioester-containing proteins, leucine-rich repeat proteins, fibrinogen-related proteins, clip-domain serine proteases, antimicrobial peptides, and pattern-recognition molecules (57, 58). However, these repertoires cannot be interpreted solely by mapping them onto *Drosophila* orthologs. Gene-family expansion creates paralogs with potential tissue-, stage-, and pathogen-specific functions, while divergence can reshape the functional organization of conserved pathways (57–59). As a result, conserved pathway labels may correspond to distinct biological outputs depending on context. Understanding how expanded gene repertoires contribute to mosquito immunity therefore requires moving beyond genome annotation alone, and instead considering the tissues and physiological states in which these molecular defenses are deployed.

The mosquito midgut is the central site for digestion and pathogen establishment, making it a key interface where physiological state and infection intersect. Although often treated as a relatively simple epithelial tube, it is in fact structurally and functionally complex. The midgut consists of three anatomically distinct regions (proventriculus, anterior, and posterior) featuring a specialized, transcriptionally dynamic epithelium that is extensively remodeled during blood feeding (32). In *Ae. aegypti*, recent transcriptomic analysis has revealed organ- and region-specific specialization, including immune-related partitioning between anterior and posterior midgut regions and large-scale transcriptional reprogramming after blood feeding (30). Similarly, in *Anopheles*, the midgut shows regional organization and responds to blood feeding and *Plasmodium* infection through coordinated digestive, barrier, redox, immune, and epithelial programs (60–64). Rather than acting as a passive site of pathogen entry, the midgut therefore functions as a dynamic physiological interface in which infection is filtered through tissue remodeling, microbiota activity, and blood-meal-induced physiological change.

In this context, local epithelial and physiological changes can generate signals that influence systemic immune activity, establishing the midgut as a central signaling hub. Mosquito immunity is therefore distributed across integrated tissues and cell populations, with hemocytes functioning as major immune sentinels. Hemocytes patrol tissues and are found both in circulation and as sessile populations associated with structures such as the midgut basal lamina, abdominal wall, Malpighian tubules, and periosteal regions (65). Their abundance, localization, and activation state can change in response to both infection-derived and physiological cues, including blood feeding (40–42, 66, 67). The mosquito immune landscape is therefore best understood as a distributed system spanning the gut epithelium, microbiota, fat body, hemocytes, and reproductive tissues, all of which can be dynamically reconfigured across physiological states.

This distributed organization has a key consequence: pathway activity measured in one compartment can shape infection outcomes at another tissue interface. Signals induced in the midgut epithelium can alter hemocyte behavior; hemocyte-derived factors can influence *Plasmodium* survival after midgut traversal; microbiota-derived cues can modulate antiviral and antiparasitic responses; and endocrine signals can coordinate reproductive physiology with immune function. These cross-compartment interactions create layered causality, in which infection phenotypes often reflect integration among local tissue state, systemic immune activity, microbiota-derived inputs, and endocrine context rather than the action of a single pathway in isolation.

## 2 The layered physiological architecture of mosquito immunity

### 2.1 The mosquito blood-fed midgut functions as a regionalized physiological-immune interface

#### 2.1.1 Regional gut physiology creates regional immune opportunity and pathogen bottlenecks

Rather than functioning as a uniform barrier, the mosquito midgut is spatially organized into distinct regions that differ in structure, physiology, and immune activity. This regionalization has direct consequences for how pathogens establish infection. Across *An. gambiae* and *Ae. aegypti*, transcriptomic and functional studies show that digestive, metabolic, barrier, and immune processes are unevenly distributed along the proventriculus, anterior midgut, posterior midgut, and hindgut-associated regions. In *Ae. aegypti*, Hixson and colleagues showed that antimicrobial peptide genes are differentially enriched along the gut axis, with the proventriculus and anterior midgut displaying distinct transcriptional enrichment compared with the posterior midgut (30). Similarly, studies in *An. gambiae* have shown region-specific diversification of immune gene expression, including peptidoglycan recognition proteins and antimicrobial peptides, between anterior and posterior gut compartments (61, 62). These patterns are consistent with functional partitioning in which distinct gut regions differ in microbial exposure, epithelial state (barrier integrity, cellular remodeling, and metabolic function), and immune capacity, creating more permissive or immune-regulated environments that may support bacterial expansion after a blood meal. As a result, pathogens encounter a sequence of spatially defined bottlenecks and immune-tolerant regions rather than a single homogeneous barrier. Successful infection therefore depends not only on pathogen traits, but also on where and when interactions occur within this structured landscape.

#### 2.1.2 Microbiota shape the immunological baseline and respond to blood feeding

Within this spatially organized system, the microbiota help establish the baseline conditions under which infection may occur. Studies in *An. gambiae* and *Ae. aegypti* have linked mosquito-associated microbial communities to development, reproduction, digestion, epithelial function, redox balance, and immune signaling. In larvae, gut bacteria are required for normal development by regulating hypoxia-related signaling, which in turn coordinates hormonal regulation, metabolic activity, and amino acid transport, ensuring proper developmental progression (51–53). Mosquito larvae acquire their microbiota from the surrounding aquatic environment, as reviewed previously (68), and community composition undergoes marked restructuring across development, with overall diversity typically declining during the transition to adulthood (69–71). However, this transition does not represent a complete reset. Instead, a subset of larval-acquired bacteria can persist into adulthood, contributing to the establishment of the adult microbiota and carrying developmental microbial history into the physiological baseline of the adult mosquito (69–71).

In adult mosquitoes, the microbiota have been linked to multiple physiological processes, including digestion, fecundity, immune activity, hemocyte recruitment and differentiation, and pathogen transmission (11, 13, 49, 55, 56, 72–76). Consistent with this, metagenomic analyses of sylvatic and domestic breeding sites have shown that environmentally acquired microbial communities can have lasting effects on mosquito physiology, such that differences in the microbial composition of larval habitats are associated with variation in adult immune status and susceptibility to viral infection (77). Thus, microbiota can establish a physiological and immunological baseline that shapes responses to blood feeding and infection. This baseline becomes particularly important after a blood meal, when nutrient influx, osmotic shifts, and altered redox conditions (resulting from simultaneous changes in gut chemistry, local immune activity, and epithelial physiology) can promote rapid bacterial expansion and ultimately reshape host-microbe interactions. Therefore, the post-blood-meal microbiota bloom reflects a transition from a pre-existing microbial and physiological state, rather than an isolated overgrowth event.

The evidence for microbiota-mediated effects on infection outcomes is strongest in systems where microbial manipulation alters pathogen success (78). In anopheline mosquitoes, midgut bacteria can restrict *Plasmodium* infection by stimulating mosquito immune responses or by directly antagonizing parasites through secreted factors (72, 79). For example, bacterial isolates from natural mosquito populations with reduced malaria transmission have revealed strain-specific antiparasitic mechanisms. Certain *Serratia* species act indirectly through activation of immune pathways such as Toll, whereas others act more directly through secreted enzymes, including lipases with antiparasitic activity (80). Similarly, *Enterobacter* species can produce reactive oxygen species that impair early parasite development (81). In *Aedes* species, microbiota also influence antiviral responses: antibiotic-mediated depletion of gut bacteria can alter viral replication (73), while colonization with specific bacterial taxa such as *Chromobacterium* or *Rosenbergiella* can reduce dengue or Zika virus transmission potential (82, 83). Naturally associated bacteria such as *Enterobacter hormaechei* can also inhibit viral entry through sphingolipids that interfere with membrane fusion, reducing susceptibility to both Zika and dengue viruses (84). Together, these studies show that variation in microbiota composition, shaped by environmental exposure and life history, can modulate vector competence either directly through microbial antagonism of pathogens or indirectly by altering mosquito immune status and broader physiological conditions.

Despite these advances, microbiota-driven phenotypes remain difficult to interpret mechanistically. Experimental approaches based on antibiotic treatment, recolonization, or community manipulation are powerful, but they can introduce effects that extend beyond microbial depletion. Antibiotic treatment and bacterial reintroduction are both perturbations of the host-microbiota system that can converge on changes in host metabolism and physiology, including nutritional status, redox balance, epithelial turnover, and barrier integrity, thereby complicating interpretation of effects on mosquito vector competence. Microbiota effects also depend not only on taxonomic composition, but on bacterial load, spatial localization, and the state of the host tissue. Bacteria confined to the blood bolus may have different consequences from bacteria in direct contact with the epithelium or populations that translocate systemically. Likewise, individual taxa may inhibit pathogens through metabolite production, immune modulation, niche competition, or alteration of the epithelial environment, whereas microbial communities can also enhance susceptibility by modifying nutrient availability or oxidative conditions. Consequently, microbiota-driven changes in vector competence should be interpreted as emergent outcomes of ecological and physiological interactions, rather than as evidence of direct immune priming alone. In this context, microbiota-derived signals can induce immune outputs that affect vector-borne pathogens independently of direct pathogen recognition, meaning that pathway activation may not always reflect pathogen-associated cues. Endosymbionts such as *Wolbachia* extend this principle from transient microbiota-derived stimulation to sustained reprogramming of host immunity, metabolism, and cellular resource allocation (85–88).

#### 2.1.3 Pathogens enter a blood-meal-remodeled landscape

**Figure 1:**
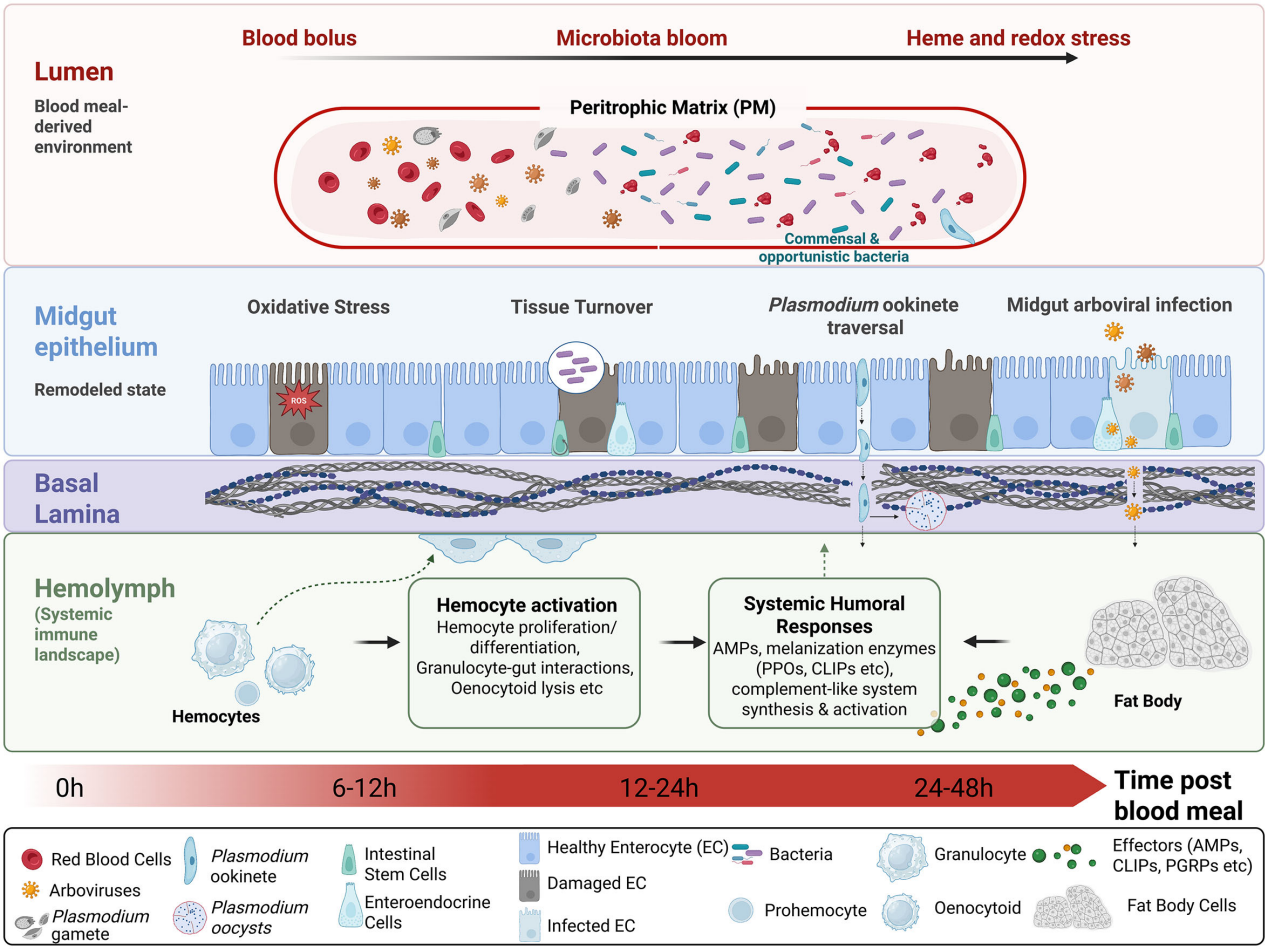
The immune landscape of mosquitoes during a blood meal. Following blood feeding, the mosquito midgut lumen undergoes major physiological and ecological shifts, including rapid expansion and subsequent restructuring of the microbiota, typically beginning around 12 h post-feeding. At the same time, blood-borne pathogens such as arboviruses or *Plasmodium* gametocytes may be introduced. Blood digestion also releases heme as a by-product of hemoglobin breakdown and induces digestive peptidases required for protein processing. During digestion, the peritrophic matrix forms as a protective barrier that separates the midgut epithelium from luminal contents and limits direct contact with microbes and pathogens. However, some parasites can bypass this barrier and interact with the midgut epithelium, which includes enterocytes (ECs), enteroendocrine cells (EEs), and stem- or progenitor-like epithelial cells. Blood feeding, heme-associated oxidative stress, and microbial interactions collectively drive epithelial remodeling and tissue turnover. In this reconfigured tissue environment, *Plasmodium* ookinetes traverse the epithelial layer, while in arbovirus-permissive mosquitoes, viruses replicate within epithelial cells and subsequently disseminate through blood-meal-associated remodeling or microperforations of the basal lamina (BL), a thin extracellular matrix underlying the epithelium. Regardless of infection status, local midgut events can generate systemic signals that are released into the hemolymph and reshape the hemocyte landscape. Together with fat body responses, these changes promote the synthesis and secretion of humoral immune factors, including antimicrobial peptides (AMPs), melanization enzymes, and complement-like proteins. These local and systemic responses shape infection outcomes in a context-dependent manner. Created in BioRender. Samantsidis, G. (2026) https://BioRender.com/46yupv1.

Arboviruses and *Plasmodium* parasites enter mosquitoes with the blood meal, but they do not encounter a static host environment. Instead, they enter a tissue landscape that is actively reshaped by digestion, epithelial immune activation, redox stress, microbiota expansion, and barrier remodeling (**Figure 1**). This framing maps naturally onto the classical sequence of vector-competence barriers, including midgut infection, midgut escape, and later salivary gland infection and escape, while emphasizing that each barrier is encountered within a changing physiological state. Thus, blood feeding imposes constraints on infection that extend beyond intrinsic pathogen biology and differ according to pathogen class, developmental stage, tissue interface, and timing.

For arboviruses, successful infection depends on midgut epithelial cell entry, intracellular replication, and evasion of antiviral defenses such as RNA interference (RNAi). These processes are strongly influenced by physiological and tissue-associated structural changes triggered by a blood meal. For instance, rapid transcriptional induction of peptidases, including trypsins, appears to facilitate dengue virus infection, as trypsin inhibitors significantly reduce viral propagation and dissemination (89). After entry into the midgut epithelium (90), the virus replicates and eventually escapes through the basal lamina (**Figure 1**). This escape is aided by blood-meal-induced restructuring of the basal lamina (33, 34, 91), an effect that becomes more pronounced with subsequent feeding events. Notably, a second, non-infectious blood meal can shorten the extrinsic incubation period for dengue, Zika, and chikungunya viruses by increasing the number of microperforations in the basal lamina, thereby accelerating dissemination (17, 92). Beyond structural remodeling, blood feeding also modulates the immune landscape by triggering the recruitment of hemocytes and altering their abundance near the midgut epithelium. These changes in hemocyte dynamics following a blood-meal may influence viral infection outcomes, although the mechanisms linking hemocyte responses to viral infections remain context-dependent. Depending on the experimental methodology, perturbations of phagocytic hemocyte function have been associated with either increased viral dissemination to secondary tissues or reduced viral spread, as discussed in more detail in section 3.3.4 (93, 94). Together, these observations indicate that arbovirus success depends on linked but distinct steps—midgut infection, basal-lamina escape, and systemic dissemination—each shaped by the blood-meal-remodeled tissue state.

In contrast, *Plasmodium* parasites are highly sensitive to the tissue remodeling that follows blood feeding. After gametocyte ingestion and fertilization, ookinetes develop within the blood bolus before traversing the midgut epithelium and establishing oocysts on the basal side (**Figure 1**). This developmental trajectory occurs within a rapidly changing environment shaped not only by the initial blood meal, but also by subsequent feeding events. In *An. gambiae*, a second blood feeding has been shown to affect parasite development in a species-specific manner: while it enhances *P. falciparum* oocyst growth, it reduces *P. berghei* development by disrupting the basal lamina and increasing parasite exposure to complement-like immune factors (95). These divergent outcomes directly implicate blood-meal-induced remodeling of the basal lamina as a determinant of immune accessibility, but they also illustrate that distinct *Plasmodium* species can navigate the same changing tissue landscape differently, with consequences for parasite survival and parasite transmission.

Within this structurally and immunologically remodeled midgut, *P. falciparum* also deploys parasite-specific mechanisms that modulate host immune detection (96, 97). Variation in parasite surface proteins, including Pfs47, has been associated with evasion of JNK-associated immune responses and suppression of epithelial nitration, thereby reducing recognition and subsequent killing in specific mosquito-parasite combinations (97–100). These effects are consistent with a lock-and-key model in which parasite haplotypes interact differentially with distinct *Anopheles* species or genotypes, producing variable outcomes in immune activation and parasite survival (101). Rather than acting independently of host physiology, these evasion mechanisms intersect with the blood-meal-induced remodeling described above, in which digestion, epithelial stress, microbiota expansion, and basal lamina restructuring shape the likelihood of immune detection. Thus, immune evasion in *P. falciparum* does not simply reflect a fixed parasite trait, but a context-dependent outcome emerging from interactions between parasite genotype, mosquito species background, and the blood-meal-dependent physiological state of the midgut.

Together, these observations do not imply that blood-meal-induced physiological remodeling affects all pathogens through identical mechanisms, nor that blood feeding elicits identical immune states across mosquito species. Rather, the consequences of this remodeled state depend on pathogen biology, tissue compartment, and the stage of infection at which host defenses are encountered. Thus, although arboviruses rely primarily on intracellular replication and are strongly influenced by RNAi-mediated antiviral restriction, *Plasmodium* parasites are more directly exposed to epithelial, redox, and complement-like defenses during tissue invasion and traversal. Yet both pathogen classes encounter a host environment that has already been extensively remodeled by the same event: blood feeding. Importantly, this blood-meal-induced physiological state does not replace pathogen-induced immunity, but instead conditions the context in which pathogen-specific responses emerge. For example, *Plasmodium* ookinete invasion induces distinct hemocyte differentiation programs through regulation of LITAF-like signaling in *An. gambiae* (47, 102), indicating that parasite invasion can generate immune responses beyond those triggered by blood feeding alone. Similarly, although blood feeding can alter hemocyte abundance and TEP1 expression (40), parasite invasion engages additional complement-associated mechanisms involving microvesicle release, TEP1 cleavage, and parasite surface deposition (48). In contrast, antiviral RNAi responses are more directly linked to the intracellular detection of viral double-stranded RNA generated during replication. However, blood-meal-induced remodeling of epithelial barriers and tissue architecture influences critical infection bottlenecks for both pathogen classes, although the consequences for pathogen survival, dissemination, and immune exposure differ across systems. These examples illustrate that pathogen restriction in mosquitoes does not occur through a single immune event, but through sequential physiological and immunological bottlenecks encountered along infection progression. Different defense layers may dominate at distinct stages, with some mechanisms arising primarily from blood-meal-induced physiological remodeling, whereas others are triggered more directly by pathogen-derived signals or tissue invasion. Thus, vector competence reflects the cumulative outcome of interactions among pathogen biology, tissue state, microbiota dynamics, and immune activation across multiple anatomical interfaces. This forms the basis for an integrated framework in which vector competence emerges from the interaction between pathogen biology and a dynamically remodeled host physiological state.

This logic also cautions against treating *Aedes* and *Anopheles* as interchangeable mosquito models. The two genera differ substantially in their physiology and immunology. For example, *Ae. aegypti* mosquitoes are more resistant to bacterial infection than *An. gambiae*, a difference that correlates with variation in immune gene architecture and transcriptional responses to infection (57, 59, 103, 104). Furthermore, *Ae. aegypti* has provided much of the experimental framework for arbovirus barriers, blood-meal-regulated midgut physiology, and *Wolbachia*-based pathogen blocking. *Anopheles* systems have provided especially detailed mechanistic insight into epithelial nitration, TEP1-centered complement-like killing, LRIM/APL proteins, CLIP proteases, melanization, and hemocyte-dependent antimalarial immunity. These systems share hematophagy and many conserved modules, but the pathogens, tissue bottlenecks, and best-supported effector mechanisms differ. Cross-species comparison is most powerful when it is used to generate mechanistic hypotheses, rather than to reduce system-specific biology to a generalized mosquito immune framework.

### 2.2 Conserved pathways acquire mosquito-specific meaning inside blood-fed tissues

#### 2.2.1 Translating the *Drosophila* immunity map to mosquito mechanisms

The canonical *Drosophila* model has provided much of the conceptual language of insect immunity. In that framework, the Toll pathway is classically associated with responses to fungi and many Gram-positive bacteria, whereas IMD responds to diaminopimelic acid-type peptidoglycan from many Gram-negative bacteria, with both pathways converging on NF-κB transcription factors and antimicrobial peptide expression (19–23). This framework remains essential, but its direct transfer to mosquitoes requires caution. Mosquitoes share many immune gene families with *Drosophila*, yet these families have undergone lineage-specific expansion, contraction, and diversification (57, 58). This diversification is observed not only between *Drosophila* and mosquitoes, but also among mosquito lineages. For example, several components of the mosquito Toll signaling pathway show lineage-specific expansions and rapid sequence divergence (105). Moreover, mosquito immune responses operate in a hematophagous context, with a midgut ecology and pathogen spectrum that differ substantially from those of *Drosophila*. Thus, conserved pathway names provide a useful map, but they do not by themselves define mechanism, tissue function, or pathogen-specific outcome in mosquitoes.

A recent comparative transcriptomic study in *Ae. aegypti* and *An. gambiae* further suggests that whole-organism transcriptional responses do not always follow the expected Gram-positive/Gram-negative pattern as in Drosophila. The authors found that mosquito transcriptional responses clearly separated fungal from bacterial infections, but did not reliably distinguish Gram-positive from Gram-negative bacteria under the tested conditions (104). This result should not be interpreted as evidence that mosquitoes lack biochemical specificity in microbial recognition. Rather, it illustrates the difficulty of inferring upstream pathway logic from downstream whole-organism transcriptional profiles. In mosquitoes, such outputs are shaped by tissue context, microbial load, route of infection, rate of damage, and physiological state, all of which can mask simple pathogen-class signatures at the organismal level.

A further consideration is that pathway activity is often inferred indirectly, which can lead phenotypes to be misinterpreted as direct evidence of pathway activation or direct pathogen sensing. Transcriptional induction of antimicrobial peptides, pathway regulators, or downstream components provides useful information, but it does not directly measure biochemical or cell-specific activation events, such as receptor engagement, NF-κB nuclear translocation, or tissue-restricted signaling dynamics. At the same time, genetic perturbation of immune pathways can influence vector competence through integrated mechanisms that extend beyond pathogen recognition, including effects on development, epithelial integrity, microbiota composition, endocrine signaling, and metabolism. Taken together, pathway–pathogen associations may therefore reflect a mixture of upstream sensing, downstream physiological effects, and indirect systemic changes rather than direct sensing alone. This distinction is important for understanding anticipatory and state-dependent immunity mechanistically because pathway conservation, pathway activation, pathway-dependent phenotype, effector or tissue-state output, and the pathogen stage affected are related but not equivalent forms of evidence.

#### 2.2.2 NF-κB signaling in mosquitoes reflects immune state rather than Gram-class recognition

**Figure 2:**
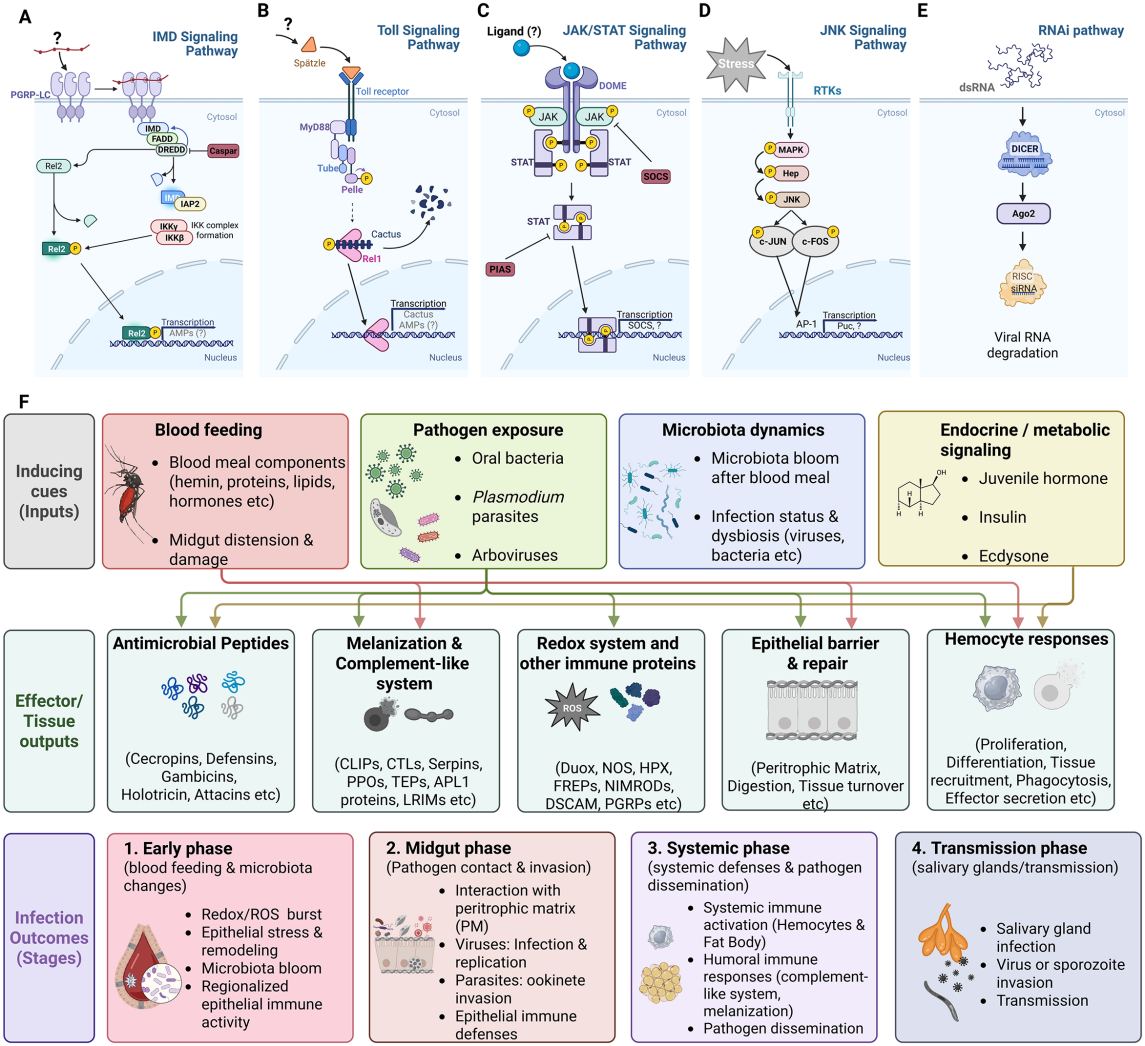
Mosquito immune modules during blood feeding and pathogen infection. **(A–E)** Schematic representation of five conserved immune modules implicated in mosquito responses to bacteria, *Plasmodium* parasites, and arboviruses. These include the **(A)** IMD/Rel2, **(B)** Toll/Rel1, **(C)** JAK/STAT, **(D)** JNK, and **(E)** dsRNA-responsive RNA interference (RNAi) pathways. **(F)** Multiple inducing cues shape mosquito immune state, including blood feeding and its downstream tissue consequences, pathogen exposure, microbiota dynamics, and endocrine signaling through ecdysone, insulin-like signaling, and juvenile hormone. These cues do not act in isolation; their effects depend on tissue context, timing, physiological state, and pathogen stage. Blood feeding can alter epithelial barrier integrity, repair processes, hemocyte activity, and melanization potential, whereas pathogen exposure can induce antimicrobial peptides (AMPs), melanization, redox responses, epithelial defenses, and hemocyte-mediated immunity. The figure organizes these interactions into four broad phases: (1) an early post-feeding phase, characterized by redox changes, localized epithelial immune activity, and microbiota expansion; (2) a midgut phase, during which pathogens encounter or invade the midgut epithelium and associated barriers; (3) a systemic phase, involving hemocytes and fat body-derived humoral immune effectors; and (4) a transmission phase, involving salivary gland infection or invasion and pathogen release into saliva, enabling transmission to a vertebrate host. Created in BioRender. Samantsidis, G. (2026) https://BioRender.com/ujamhgt.

Toll and IMD pathways clearly influence mosquito susceptibility to microbes and pathogens, but their functional roles are strongly context-dependent. Transcriptomic analysis of *Ae. aegypti* has shown that dengue virus infection activates the Toll signaling pathway (106). The functional significance of this pathway was further resolved by experimentally activating it by silencing the Toll inhibitor Cactus (**Figure 2B**), which reduced infection rates, whereas pathway suppression increased susceptibility (73, 107). However, cell-based assays have shown that exposure to viral particles alone is insufficient to induce Toll signaling (108). This discrepancy suggests that Toll pathway activation *in vivo* may not result from direct viral recognition, but instead reflects additional layers of regulation present in the whole organism. These may include microbiota composition, tissue context, or physiological state. Moreover, flavivirus infection itself can alter the composition of the mosquito microbiota, potentially favoring or suppressing bacterial taxa that could indirectly contribute to pathway activation (109, 110). Together, these observations indicate that while Toll pathway activity shapes dengue infection outcomes, it does not necessarily reflect direct activation of a canonical Toll recognition cascade. Instead, Toll signaling may regulate a basal antiviral or epithelial state that alters the likelihood of successful infection.

A similar pattern emerges across bacterial, fungal, and parasitic systems. Early work showed that Toll and IMD pathways contribute to antibacterial and antifungal defenses (111–113), and both pathways have been implicated in regulating *Plasmodium* infection in *Anopheles* mosquitoes (114–117). However, these studies also show that pathway function cannot be reduced to a simple microbial-class assignment. For example, Toll signaling appears particularly important for controlling *P. berghei*, whereas IMD signaling has been more strongly associated with resistance to *P. falciparum*, in part through regulation of complement-like factors such as APL1 family proteins (118). At the same time, combinatorial perturbation experiments demonstrate substantial overlap and redundancy: simultaneous disruption of Toll and IMD signaling can limit *P. berghei* development through effects on key antiparasitic factors such as LRIM1 and TEP1, while single-pathway perturbations have limited effects (114). Manipulation of pathway regulators further complicates a linear interpretation of NF-κB signaling. Silencing the Toll inhibitor Cactus leads to strong upregulation of antiparasitic effectors and a marked reduction in parasite burden (114). However, this effect cannot be explained solely by direct Toll-dependent pathogen recognition. Although Cactus silencing induces substantial transcriptional changes, infection further enhances the expression of genes like CTL4, TEP1, or LRIM1 rather than producing the expected plateau (114). This indicates that infection does not simply recapitulate the Cactus-induced transcriptional state and that the regulation of these genes integrates additional inputs, potentially including microbiota-derived signals associated with dysbiosis or broader physiological changes (114). Alternatively, systemic pathway activation may alter microbiota composition or host metabolic state, creating conditions that indirectly limit parasite development rather than directly enhancing immune recognition.

The Rel2/IMD arm (**Figure 2A**) illustrates the same problem at the level of pathway state and microbiota homeostasis. Although Rel2-dependent responses contribute to antibacterial defense and antiparasitic immunity, recent work reported in a bioRxiv preprint shows that REL2 overexpression in the *An. gambiae* midgut can drive extensive transcriptional reprogramming without producing a straightforward canonical immune response against *P. falciparum* (119). This distinction matters because overexpressing a transcription factor is not equivalent to receptor-driven pathway activation during microbial exposure, infection, or blood feeding. In parallel, IMD signaling has been identified as a key regulator of microbiota homeostasis, with disruption of receptors such as PGRP-LC leading to increased bacterial proliferation after blood feeding (79, 120). Thus, IMD signaling may influence parasite infection not only through direct antiparasitic effectors, but also by modulating the microbial and epithelial environment in which parasite development occurs (**Table 1**).

**Table 1:** Evidence for anticipatory immunity and related immune-state categories in *Anopheles* and Aedes mosquitoes.

Category	Initiating cue(s)	Main tissue/ physiological context	Mechanistic interpretation	Representative systems/examples	Evidence status/limitation	Key references
Blood-meal-induced immune readiness (Immune-relevant physiological conditioning triggered by blood feeding before, or in parallel with, pathogen-specific cues)	• Blood ingestion • Heme release from hemoglobin digestion • Midgut distension • Digestive and redox remodeling • Blood-meal-associated basal lamina and peritrophic-matrix remodeling • Post-feeding hemocyte activation	• Midgut lumen and epithelium • Peritrophic matrix • Basal lamina • Hemocytes • Hemolymph/fat-body effector availability	Modifications in barrier integrity, epithelial state, redox balance, and humoral immunity that alter defense responses and tissue permissiveness.	• *An. gambiae*: non-infectious blood feeding induces LRIM9, an anti-*Plasmodium* factor, independently of direct parasite induction. • *An. gambiae*: blood feeding induces hemocyte proliferation/activation. • *An. gambiae*: additional blood feeding events change basal lamina architecture leading to parasite species-specific responses. • *Ae. aegypti*: blood-meal-induced basal lamina remodeling/microperforation can accelerate arbovirus dissemination. • *Ae. aegypti*: blood/heme-dependent HPx1 affects peritrophic matrix/redox state, microbiota expansion, and arbovirus permissiveness.	Direct experimental support exists in defined mosquito–pathogen systems. Blood-meal-induced physiological remodeling can be protective, neutral, or permissive depending on pathogen, tissue interface, and infection stage. Generalization across mosquito genera, pathogen classes, and infection stages remains inferential.	(17, 33, 34, 40, 45, 67, 92, 93, 141, 165)
Microbiota-driven immune state (Immune, epithelial, metabolic, and redox state shaped by resident microbiota composition, bacterial load, spatial localization, and post-blood-meal microbial expansion)	• Resident gut microbiota • Post-blood-meal microbiota bloom • Larval/ environmental microbial carryover • Microbial products/ metabolites • Wolbachia or other persistent symbiont effects	• Midgut lumen and epithelium • Local redox/barrier environment • Fat body and systemic immune tone • Hemocyte recruitment/ differentiation in selected host-pathogen systems	Immune pathway activation, direct pathogen antagonism, enzyme secretion, ROS/metabolite production (sphingolipids), epithelial conditioning and barrier modification.	• *An. gambiae*: midgut bacteria restrict *Plasmodium* via immune stimulation and/or direct antagonism; PGRP-LC/IMD controls bacterial homeostasis and affects parasite infection. • *Enterobacter* isolates can produce ROS that impair early development of *Plasmodium* in *Anopheles*. • *Serratia* and other taxa can influence *Plasmodium* development through immune-dependent (Toll) or secreted-factor mechanisms. • *Ae. aegypti*: microbiota manipulation alters dengue/Zika/Sindbis outcomes; *Chromobacterium*, *Rosenbergiella*, and *Enterobacter hormaechei* show direct or indirect antiviral mechanisms. • *Ae. aegypti* oral bacterial infection induces epithelial JAK/STAT signaling associated with regeneration and host survival without reduced bacterial burden (preprint).	Direct evidence exists for specific bacterial taxa, experimental manipulations, and host–pathogen systems. However, antibiotic treatment, recolonization, and microbiota perturbation can also alter host metabolism, redox balance, barrier integrity, and epithelial turnover. Consequently, microbiota-dependent phenotypes should not automatically be interpreted as immune priming, but may instead reflect broader and indirect effects on vector competence through alterations in host physiology. Midgut infection induces the activation of an Upd-like/JAK/STAT signaling promoting midgut repair/tolerance-associated phenotype: direct pathogen restriction not demonstrated	(13, 72–87, 125)
Endocrine/metabolic priming (Immune modulation by endocrine, nutritional, and blood-derived signals coupled to reproductive and metabolic physiology)	• Pre blood meal: Juvenile hormone • Early blood meal: Vertebrate-derived insulin, IGF1, and TGF-β1 • Post-blood meal: 20-hydroxyecdysone (20E) • Post-blood meal: Insulin/insulin-like signaling (IIS) and TOR	• Fat body • Midgut epithelium • Hemocytes/ oenocytoid-linked effector arms • Ovaries/ reproductive tissues • Blood bolus as signaling & effector environment	Coordinated reproduction, nutrition, oxidative stress, epithelial state and immune activity (hemocyte abundance, AMP expression, humoral factors). Outcomes may vary across mosquito species, time, and tissue.	• *An. gambiae*: 20E induces expression of immune-associated genes (PPO/CLIP/AMPs etc). Primed immunity against bacteria and *P. berghei*. • *Ae. aegytpi*: 20E induces expression of the EcR-regulated Pirk, which inhibits IMD signaling in mosquito abdominal tissues. • *Ae. aegypti*: JH regulates AMP and immune/metabolic gene expression in the midgut and abdomen. JH can alter susceptibility to ZIKV. • *Anopheles*: IIS and vertebrate blood factors modulate NOS/MAPK/NF-κB, oxidative stress, lifespan, and *Plasmodium* development.	Direct experimental evidence exists for selected endocrine axes, but their effects are highly context dependent. 20E can enhance immune activity in some systems while suppressing it in others, and IIS/JH signaling can be permissive, suppressive, or restrictive depending on tissue, physiological state, and infection context.	(35–37, 42, 67, 146–149, 164, 166–171, 173, 174, 176–179)
Infection-induced immune priming (Enhanced immune responsiveness generated by prior infection, pathogen-induced tissue invasion, damage, or microbial translocation)	• Prior *Plasmodium* exposure (prostaglandins/lipoxin/lipocalin-associated signaling) • Bacterial challenge • Ookinete-induced epithelial disruption • Microbiota contact with injured midgut cells	• Midgut epithelium • Hemocytes • Hemolymph (humoral mediators)	Prior infection or tissue damage can generate a long-lasting immune state characterized by hemocyte differentiation and lipid mediator signaling (e.g., Evokin/lipoxin-associated HDF activity, Prostanglandins), resulting in enhanced resistance upon secondary challenge.	• *An. gambiae–P. berghei*: Hemocyte differentiation and enhanced secondary anti-*Plasmodium*/antibacterial responses. • *An. gambiae–P. berghei*: Microbiota-dependent HDF (Evokin/lipoxin) and prostaglandin-mediated systemic immune priming.	Direct experimental support is strongest for *An. gambiae–Plasmodium* and selected bacterial-challenge systems. Evidence for analogous mechanisms in Aedes-arbovirus interaction systems or across mosquito genera is limited.	(75, 76, 161)
Pathogen-specific immune responses (Responses induced more directly by pathogen-derived molecules, replication intermediates, pathogen-stage-specific invasion, or pathogen-associated tissue damage)	• Viral double-stranded RNA replication intermediates • *Plasmodium* ookinete invasion • Parasite surface proteins/haplotypes • Epithelial nitration/apoptosis cues • Complement-like parasite recognition	• Virus-infected midgut and systemic cells • *Plasmodium*-traversed midgut epithelium • Hemolymph/fat body/hemocyte-derived complement-like effectors	Pathogen-derived or pathogen-induced tissue-specific cues engage immune mechanisms (IMD, Toll, JAK/STAT, JNK, RNAi) and activate both cellular (hemocytes) and humoral (complement-like system, melanization associated enzymes etc) defenses. These responses may still be modulated by host physiological state.	*• Ae. aegypti* – arboviruses: mosquito RNAi is linked to viral dsRNA and affects arbovirus replication/dissemination (Loqs2 controls DENV/ZIKV replication in tissue-dependent ways). *• Ae. aegypti* DENV infection induces JAK/STAT-responsive genes (e.g. SOCS, DVRF1, DVRF2) in midguts; fat body-specific Dome/Hop overexpression restricts DENV. • *Anopheles*–*Plasmodium*: Pfs47-mediated immune evasion intersects with JNK-associated apoptosis/nitration and mosquito genotype. • *Anopheles*: TEP1/LRIM/APL and CLIP-protease modules mediate complement-like parasite killing or melanization. • *Anopheles*-*Plasmodium* systems show JAK/STAT-associated antiparasitic phenotypes	Direct experimental evidence exist in both vector-pathogen interaction systems, where inducing pathogen-associated cues and effector outputs can be linked. For JAK/STAT pathway activation, evidence exists in *Ae. aegypti* in viral infection models, but only limited information is available in *Anopheles* host-pathogen systems. The pathogen-specific responses are shaped by blood feeding, microbiota, tissue state, and mosquito/pathogen genotype.	(97–101, 128, 129, 131–134, 138–140, 143–145)

Variation across experimental systems further reinforces this interpretation. Differences in microbiota composition, mosquito genetic background, parasite strain compatibility, and experimental design can produce divergent phenotypes, even when the same pathway components are targeted. These observations indicate that NF-κB pathway outputs are shaped by activation modality, tissue context, microbiota composition, and physiological state. They also emphasize that experimentally imposed transcription factor activity does not necessarily recapitulate the biological state produced by receptor-driven pathway activation during natural infection or blood feeding, when pathway induction occurs alongside feedback regulation and homeostatic constraints. Together, these findings support a model in which mosquito NF-κB pathways regulate immune state rather than simply assigning microbial classes to Toll or IMD. This state encompasses antimicrobial effector expression, epithelial integrity, microbiota control, and broader physiological conditions that influence pathogen survival. Resolving the mechanistic roles of these pathways will therefore require pathway-resolved measurements in relevant tissues and physiological contexts, including direct assays of Rel1 and Rel2 activity, cell-type-specific transcriptional outputs, microbiota-controlled experiments, and readouts that distinguish direct pathogen killing from altered host permissiveness.

#### 2.2.3 Context-dependent function of mosquito JAK/STAT signaling

The JAK/STAT pathway (**Figure 2C**) illustrates both the strength and the interpretive limits of pathway-centered descriptions. In *Ae. aegypti*, dengue virus infection has been associated with activation of JAK/STAT-related transcriptional responses, including SOCS and other STAT-dependent effector outputs, demonstrating pathway activation during infection (121). The same study also identified two putative antiviral effectors, dengue virus restriction factor 1 and 2 (DVRF1 and DVRF2), whose RNAi-mediated silencing increased dengue viral loads, supporting a potential mechanism through which JAK/STAT signaling contributes to viral restriction (121). In addition, experimental overexpression of Dome, the JAK/STAT receptor, or Hop, the Janus kinase homolog, reduced mosquito permissiveness to dengue and ZIKV infection and increased expression of DVRF1 (122). In the same study manipulation of JAK/STAT signaling did not influence pathogen loads following bacterial infection or infection with Zika or Chikungunya virus.

In these two studies, although experimental manipulation demonstrates that JAK/STAT activity can influence infection outcomes, it remains necessary to identify the endogenous inducing signal, responding tissue, downstream effector program, and pathogen stage affected during natural infection. A useful comparison comes from recent preprint-based evidence showing that oral bacterial infection of the *Ae. aegypti* midgut induces an Upd-like/JAK/STAT signaling module in the epithelium that promotes epithelial regeneration and improves mosquito survival without affecting the bacterial burden (123). This study recapitulates the limited antibacterial activity of the pathway previously observed (122) and places JAK/STAT signaling closer to epithelial repair and host tolerance.

A useful way to understand JAK/STAT associated phenotypes is to parse them into pathway activation, engineered activation and restriction, epithelial repair, tolerance, pathogen restriction, and altered permissiveness and all of those represent different biological outcomes. Here, the term tolerance is used in the strict infection-biology sense: preservation of host fitness or reduced damage at a given pathogen or microbial burden, independently of pathogen clearance. Epithelial repair refers to restoration of tissue integrity following damage. Altered permissiveness describes changes in the physiological state of tissues that influence pathogen establishment, replication, or dissemination, without necessarily affecting pathogen killing. By contrast, pathogen restriction refers to a measurable reduction in pathogen burden. These outcomes may occur together in some systems but should not be considered interchangeable consequences of JAK/STAT activation.

The interpretation of antiviral and antiparasitic JAK/STAT phenotypes therefore requires caution. For example, Dome or Hop overexpression substantially reduces reproductive output in *Ae. aegypti* (122), indicating that JAK/STAT manipulation can alter broader physiological and metabolic processes. Likewise, JAK/STAT-dependent changes in tissue repair, epithelial homeostasis, or metabolic state may indirectly influence pathogen susceptibility by modifying tissue permissiveness. Thus, reduced infection levels following pathway activation may not necessarily imply direct antimicrobial effector activity.

The anti-*Plasmodium* function of JAK/STAT signaling provides an additional cautionary example. The pathway has been implicated in late-phase immunity in *An. gambiae* and early control of *P. vivax* in *An. aquasalis* (124, 125). However, pathway activity can influence parasite development through mechanisms that may be at least partly independent of the classical complement-like system, potentially involving epithelial homeostasis as well as immune effector regulation. Although JAK/STAT is associated with pathogen restriction in multiple contexts, the inducing signals, responsive tissues, and downstream effects can vary substantially depending on whether the stimulus is viral, bacterial, parasitic, or physiological in origin. Thus, a single pathway label can encompass multiple, partially distinct effector layers.

#### 2.2.4 JNK, inflammatory, and complement-like circuits extend pathway logic to tissue interfaces

This distinction becomes clearer when considering inflammatory circuits that operate in parallel with canonical pathway modules. In *Anopheles*, JNK signaling (**Figure 2D**) has been associated with apoptosis and nitration of invaded epithelial cells, contributing to parasite recognition by humoral immune factors (18, 99, 100). Here, the relevant immune phenotype is not confined to a single effector, but emerges from coordinated epithelial stress responses, redox regulation, and parasite evasion strategies. Other inflammatory mediators, including TNF-like signaling and prostaglandins, illustrate how local immune circuits can coordinate systemic state across tissues. In *An. gambiae*, TNF-like signaling has been implicated in cellular immune responses associated with parasite killing (126), while prostaglandin E2 signaling regulates hemocyte recruitment to the midgut basal lamina and oenocytoid lysis, thereby influencing both bacterial and *Plasmodium* survival (75, 127). These pathways are especially relevant in the blood-feeding context, where local midgut events can propagate systemic changes in immune readiness, linking tissue-level responses to organism-wide modulation of immune state.

Complement-like immunity further highlights how conserved functional labels can conceal substantial context dependence. In *An. gambiae*, TEP1 and associated LRIM/APL1 family proteins play central roles in *Plasmodium* killing and are major determinants of parasite survival in the mosquito (**Table 1**) (38, 128, 129). However, their activity is shaped by mosquito genetic background, parasite species, and evasion mechanisms, and does not translate uniformly across all mosquito–parasite combinations (98, 100, 129, 130). For instance, mechanisms that are effective against *P. berghei* may differ in relevance or magnitude during *P. falciparum* infection, reflecting differences in host–parasite compatibility and immune evasion strategies (101).

Together, these inflammatory and complement-like circuits reinforce a common principle: conserved pathway labels such as JAK/STAT, Toll, IMD, JNK, and complement-like immunity identify molecular modules, but not their functional meaning in a given infection. That meaning depends on the inducing cue, the responding tissue, the effector layer engaged, and the pathogen stage being affected. Infection phenotypes are therefore better understood not as outputs of single pathways, but as tissue-state outcomes shaped by epithelial stress, redox regulation, hemocyte recruitment, circulating effector availability, and host–pathogen compatibility. This framework does not diminish the value of pathway analysis; it specifies what pathway analysis must be linked to in order to explain vector competence.

#### 2.2.5 Antiviral RNAi is a direct defense embedded in a changing tissue state

Small-RNA pathways, particularly the exogenous siRNA pathway, represent major antiviral defenses in mosquitoes and are central to arbovirus–vector interactions (131–134). Unlike inducible NF-κB signaling frameworks, RNA interference is directly coupled to viral replication intermediates and generates sequence-specific antiviral activity. In the framework developed here, RNAi therefore occupies a distinct position: it is not primarily triggered by blood feeding or microbiota, but its effectiveness is nevertheless shaped by cell state, viral replication dynamics, tissue barriers, and prior physiological conditions.

Mechanistically, the siRNA pathway detects viral double-stranded RNA, which is processed by Dicer-2 into small interfering RNAs that guide Argonaute-2-mediated cleavage of viral genomes (**Figure 2E**) (131, 132). Piwi-interacting RNA pathways can also contribute to persistent arbovirus infections (135). However, RNAi activity does not translate into uniform viral clearance. Instead, mosquitoes frequently sustain persistent, non-lytic infections while retaining transmission capacity. Outcomes are influenced by viral replication kinetics, tissue tropism, sequence diversity, and the production of viral RNA species that can modulate RNAi efficiency. In this regard, some flaviviruses produce subgenomic RNAs that can interfere with RNAi-associated antiviral activity, highlighting that viruses actively shape the RNAi environment rather than simply serving as passive targets (136, 137). A useful mechanistic refinement comes from the dsRNA-binding protein Loqs2 (**Table 1**). In *Ae. aegypti*, low endogenous Loqs2 expression in the midgut is associated with permissiveness to viral proliferation, whereas Loqs2 is required to control systemic replication of dengue and Zika viruses (138). This makes Loqs2 a particularly clear example of a host factor that directly influences RNAi-mediated antiviral activity, because virus-derived double-stranded RNA serves as an immediate substrate of the pathway. Even in this relatively direct antiviral system, the resulting phenotype remains dependent on tissue context and infection stage. Effects measured at the level of midgut infection, epithelial escape, or systemic dissemination represent related but distinct barriers, and altered RNAi efficiency at one step does not necessarily translate uniformly across all stages of viral spread.

This distinction is important because RNAi operates within a tissue environment that is dynamically shaped by blood feeding, microbiota, and systemic physiology. As a result, RNAi-mediated antiviral control is influenced by infection stage and tissue context, particularly across the midgut and systemic phases of infection. Accordingly, antiviral RNAi should not be viewed as an isolated determinant of vector competence, but as a direct defense operating within a shifting physiological and tissue landscape shaped by feeding history and host state.

#### 2.2.6 Effector modules reveal the gap between pathway labels and immune mechanisms

If pathway labels describe conserved signaling modules, effector systems reveal how context-dependent their biological outputs can be. In mosquitoes, immune pathways converge on a set of immune effectors and recurrent processes, including antimicrobial peptides, reactive oxygen and nitrogen species, melanization cascades, complement-like factors, phagocytosis, apoptosis, and epithelial turnover (**Figure 2F**). These processes should not be treated as interchangeable readouts of pathway activity. A single upstream perturbation can shift several effector layers simultaneously, while the same effector class can serve different functions depending on tissue, timing, and pathogen stage. As the examples below illustrate, assigning an infection phenotype to a pathway requires identifying which effector process is altered, where it acts, and how it changes pathogen survival or host permissiveness.

One clear example comes from redox and barrier biology in the midgut. Rather than functioning solely as antimicrobial weapons, reactive oxygen species and peroxidase-linked systems are partitioned across distinct roles in time, tissue, and pathogen stage. In *Anopheles*, the IMPer/HPX15–DUOX system contributes to a dityrosine matrix that spatially separates the microbiota from the epithelium and limits immune activation, whereas HPx2/NOX5-associated nitration acts at the *Plasmodium* traversal interface to promote recognition by downstream immune factors (99, 139, 140). Similarly, in *Ae. aegypti*, HPx1 links blood/heme responsiveness with peritrophic matrix function, redox control, microbiota expansion, epithelial proliferation, and arbovirus susceptibility (141). These examples illustrate that “redox immunity” is not a single functional output, but a set of stage- and location-specific processes that can enforce barrier homeostasis, promote antiparasitic recognition, or alter viral permissiveness.

A second example is the *Anopheles* complement-like system. TEP1 is often summarized as a mosquito complement-like factor, but the functional module is broader than TEP1 alone. It includes stabilization and specificity factors such as LRIM1 and APL1C, parasite-binding and regulatory steps, and CLIP-domain protease cascades that influence whether parasites undergo lysis, melanization, or survival, in a manner that is shaped by the specific host-parasite combination and infection context (45, 58, 118, 129, 142–145). This system is mechanistically richer than a pathway label. It is produced largely by hemocytes or fat body, operates in the hemolymph, acts on parasite stages after midgut traversal, and is shaped by epithelial signals produced by invaded cells. A phenotype involving TEP1 is therefore not simply a Toll, IMD, or JNK phenotype, but it reflects an interface between epithelial damage or recognition, circulating effector availability, parasite stage, and proteolytic execution.

Taken together, these examples reinforce the idea that effector systems do not simply mirror the activation of a particular pathway. Instead, they define the functional interface at which signaling events, tissue physiology, and pathogen developmental stage are translated into pathogen restriction, tissue repair, tolerance, or altered permissiveness. Accordingly, pathway perturbations should often be interpreted not only as changes in upstream signaling, but also as changes in effector availability, tissue state, and the timing of pathogen exposure to those effector layers. This point is central for blood-fed mosquitoes, because many of the tissue and effector states that shape infection outcomes are already being built by the blood meal before the pathogen becomes the dominant signal.

### 2.3 Blood feeding builds immune state before pathogen-specific cues dominate

#### 2.3.1 The blood meal creates barrier, redox, and microbial challenges at once

Upon ingestion of a blood meal, mosquitoes undergo extensive physiological and immunological remodeling even in the absence of pathogens. Blood feeding distends the midgut, alters osmotic and metabolic conditions, modulates digestive enzyme expression, introduces heme-associated oxidative stress, and creates a nutrient-rich environment that supports expansion of the resident microbiota (24, 30, 31). These processes unfold over a time window that overlaps with key stages of arbovirus replication and *Plasmodium* ookinete traversal in the midgut of *Aedes* and *Anopheles* mosquitoes, respectively (**Figure 1**). As a result, infection phenotypes measured after an infectious blood meal likely reflect the combined influence of pathogen-driven responses and blood-meal-induced remodeling of epithelial barrier state, redox balance, microbial load, and immune effector availability.

In addition to mosquito-derived responses, the blood meal can introduce biologically active vertebrate factors that remain functional within the mosquito midgut. These molecules may vary with the physiological, immune, or metabolic state of the vertebrate host and can influence infection outcomes through distinct routes. For example, vertebrate-derived insulin and TGF-β1 have been shown to modulate mosquito nitric oxide synthase and MAPK/NF-κB activity, with downstream consequences for *Plasmodium* development (146–149). Other blood-borne components, including antibodies targeting parasite sexual stages, can also remain active within the mosquito midgut and reduce malaria transmission (142, 150). Together, these observations indicate that the blood meal is not only a nutritional substrate, but also an exogenous signaling and effector environment that can shape infection outcomes through mosquito-mediated physiological responses or direct activity within the blood bolus.

Heme provides a concrete example of how a blood-derived component can organize the post-feeding midgut environment. Rather than acting only as a metabolic byproduct or oxidative stressor, heme can influence barrier and redox conditions that affect subsequent infection. In *Ae. aegypti*, the peroxidase HPx1 is induced in response to heme and contributes to peritrophic matrix assembly and redox regulation; perturbation of HPx1 alters ROS levels, microbiota expansion, and ultimately vector competence (141). This example illustrates how a blood-meal-derived input can establish epithelial and microbial conditions that influence the likelihood of infection, dissemination, or transmission. Such effects may be overlooked when phenotypes are interpreted solely in terms of direct pathogen interactions or canonical pathway activation.

This state dependence explains why the same genetic perturbation can carry different meanings across experimental contexts. Silencing an immune gene may alter the microbiota bloom, gut redox balance, epithelial turnover, or hemocyte-derived effector availability before it changes the fate of an arbovirus or parasite. Conversely, a blood-meal-induced output may restrict infection without having been induced by the pathogen itself. In many cases, therefore, the measured phenotype identifies an altered infection outcome more clearly than it identifies the initiating cue. Accounting for this temporal order is essential: perturbation effects may reflect pre-existing changes in microbial load, epithelial barrier or redox state, tissue repair, or systemic effector availability, whereas pathogen-associated restriction can arise from responses built by feeding. Distinguishing inducing cues from downstream effects is therefore central to interpreting variability across studies and to separating resistance, repair, tolerance, and altered permissiveness.

#### 2.3.2 Microbiota bloom as a physiological immune input rather than a passive target

Blood feeding frequently expands midgut bacterial loads, but the resulting microbiota bloom is not a single variable. Its magnitude, taxonomic composition, and spatial localization vary with colony, diet, environment, antibiotic treatment, and mosquito species (77, 81, 151). These dimensions should be separated analytically. Microbial burden can drive dose-dependent immune or stress responses; community composition can determine whether metabolites, secreted effectors, or competitive interactions restrict or favor infection; and localization determines whether bacteria remain confined to the bolus, contact the epithelium, or escape into systemic tissues. The microbiota can protect against pathogens through competition, metabolite production, immune priming, redox effects, or induction of antimicrobial factors. It can also promote infection by damaging barriers, altering digestion, changing metabolites, or increasing stress responses. Microbiota effects on vector competence therefore should not be reduced to a simple protective or permissive label.

**Figure 3:**
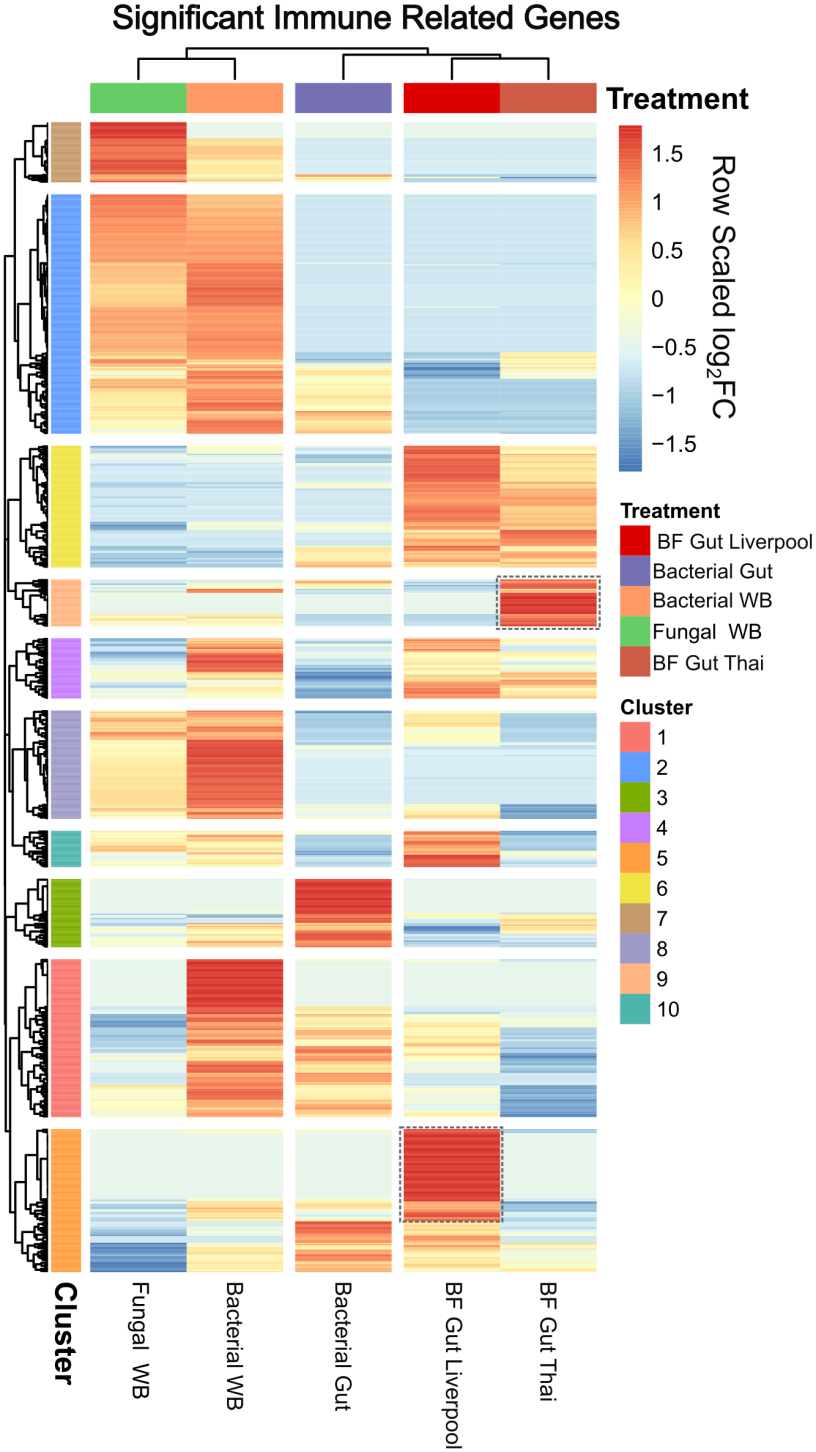
Blood feeding and microbial infection induce distinct, partially overlapping immune transcriptional signatures. A two-way hierarchical clustering of immune-gene expression across five published datasets identified ten clusters of differentially regulated immune genes in microbe-challenged mosquitoes and blood-fed midguts. Datasets include gut transcriptomes from mosquitoes orally infected with bacteria (123), whole-body (WB) transcriptomes from mosquitoes infected with bacteria or fungi (104), and blood-fed gut transcriptomes from Thai (30) and Liverpool (31) *Ae. aegypti* strains. The immune-gene catalog was derived from previous work (104). Differential expression was determined using DESeq2 with thresholds of log2FC > 1.5 and adjusted *p* < 0.05. Colors represent row-scaled log2 fold change relative to the appropriate sugar-fed or unchallenged controls. This heatmap was generated from published differential-expression outputs, as described in the **Supplementary Methods**. Hierarchical clustering was performed on the processed log2 fold-change matrix using the pheatmap package in R. Log2 fold-change values were row-scaled (Z-score normalization) prior to clustering. Rows (genes) and columns (experimental conditions) were clustered using Euclidean distance and complete linkage. The resulting gene dendrogram was partitioned into 10 clusters, and the condition dendrogram was partitioned into 3 clusters. Dataset metadata, immune-gene annotations, processed values, and cluster assignments are provided in **Supplementary Tables 3**–**S5**, and the visualization script is provided as additional file (github). A summary of immune gene categories showing condition-specific and shared responses across the different datasets is provided in **Supplementary Table 1**. A list of genes summarizing strain-specific immune gene responses to blood-feeding is included in **Supplementary Table 2**.

In this context, immune-gene expression or pathway-associated outputs detected after blood feeding may reflect bacterial expansion, tissue stress, or their interaction, even when the most visible phenotype is associated with a co-infecting virus or parasite. This complicates interpretation, particularly in experimental systems where microbiota composition or abundance differs between strains or species. To examine both divergence and overlap, **Figure 3** synthesizes published transcriptional datasets under a common immune-gene framework, using a previously curated list of immune genes from our group (104) to compare responses to non-infectious blood meals across two *Ae. aegypti* strains, Liverpool and Thai, with responses to oral or systemic bacterial infection and fungal infection (30, 31, 104, 123). Several immune-gene classes were shared across feeding and infection contexts, including antimicrobial peptides (AMPs), CLIP-domain serine proteases, serpins, and C-type lectins (**Supplementary Table 1**). At the same time, distinct gene clusters were preferentially induced by systemic infection, oral infection, or blood feeding. Blood feeding in particular was associated with modulation of genes involved in pathogen recognition, such as members of the leucine-rich repeat protein families, scavenger receptors, and Gram-negative binding proteins (GNBPs) (**Figure 3**; **Supplementary Table 1**). Comparisons between the two mosquito strains further suggested variation in blood-meal-responsive gene sets (**Figure 3**; **Supplementary Table 2**), which may reflect differences in genetic background, laboratory history, associated microbiota, or their combination. This analysis does not determine which blood-meal-responsive genes are induced independently of the microbiota. Its value is more specific: it shows that blood feeding and microbial infection induce distinct but partially overlapping immune transcriptional programs, with context- and strain-specific signatures.

Together, these observations indicate that blood feeding and the associated microbiota bloom help establish a midgut immune state that is distinct from, but partially overlapping with, immune programs associated with bacterial or fungal infections. This state is not defined simply by immune-gene induction, but by linked changes in microbial load and localization, epithelial stress, redox balance and barrier function, and effector availability. It provides the physiological and molecular context in which arboviruses and parasites encounter the gut and should therefore be considered when interpreting infection phenotypes and their underlying mechanisms. A synopsis of the microbiome’s influence on mosquito innate immunity is provided in **Table 1**.

#### 2.3.4 Epithelial remodeling links tissue homeostasis to pathogen permissiveness

Mechanistic understanding of epithelial dynamics in the mosquito midgut remains limited by the lack of genetic tools for labeling, lineage tracing, and tracking specific epithelial cell populations *in vivo*. Despite this limitation, recent work in *An. gambiae* and *Ae. aegypti* has revealed that blood feeding and infection can induce substantial remodeling of the midgut epithelium. These findings suggest that epithelial structure, turnover, and barrier function are dynamic physiological traits rather than static properties of the mosquito midgut.

In *Ae. aegypti*, blood feeding induces a relatively modest increase in epithelial turnover, reflected by elevated mitotic activity and DNA synthesis measured using phospho-Histone H3 (PH3) and EdU incorporation (32). By comparison, blood-fed *Anopheles* midguts display a stronger shift toward endoreplication, suggesting that epithelial maintenance after feeding can involve species-dependent combinations of proliferation and increased DNA content. Oral bacterial infection induces mitotic proliferation in both mosquito systems, consistent with epithelial renewal as a homeostatic response to tissue damage. Infection also increases the proportion of cells with elevated DNA content, suggesting that epithelial cells may subsequently enter differentiation or tissue-replenishment programs as part of repair after damage (32, 152, 153).

Although these observations support a model in which epithelial renewal contributes to tissue homeostasis, infection-specific epithelial responses remain poorly resolved at the cellular level. A recent study using a transgenic *An. stephensi* line that fluorescently labels hemocytes and midgut cells with stem- or progenitor-like characteristics provided new insight into epithelial responses during infection with the rodent malaria parasite *P. berghei* (154). In this model, *P. berghei* infection induced epithelial reprogramming associated with increased numbers of labeled epithelial cells during early oocyst development, rather than during ookinete invasion. The study further implicated JAK/STAT signaling in regulating both parasite survival and the proliferative behavior of these labeled epithelial cells (154). Interestingly, these cells extended pseudopodia-like projections toward developing parasites, although the mechanism and functional consequence of these interactions remain unclear. Epithelial remodeling, however, is unlikely to be regulated solely by epithelial-intrinsic programs. The mosquito midgut communicates continuously with circulating and sessile hemocytes, and recent evidence shows that phagocytic cell depletion compromises gut barrier integrity and alters permissiveness to *P. falciparum* infection (155). Together, these findings suggest that epithelial renewal and barrier maintenance emerge from coordinated interactions between epithelial and immune cell populations, reinforcing the idea that midgut physiology is embedded within a broader systemic immune context.

A potential role for epithelial-state pathways in vector competence has also been suggested in *Ae. aegypti*, where perturbation of Notch signaling, a pathway associated with intestinal stem cell differentiation in *Drosophila* (20), altered susceptibility to dengue virus infection only in a refractory mosquito strain (156). This strain-dependent effect suggests that pathways associated with epithelial maintenance and differentiation can acquire distinct functional consequences depending on tissue state, timing, and infection context. Together, these findings support the view that epithelial remodeling is not merely a passive consequence of blood feeding or infection, but a dynamic physiological process that can influence tissue permissiveness and pathogen establishment.

#### 2.3.5 Hemocytes integrate gut-derived signals into systemic immune responses and infection outcomes

Hemocytes are central components of mosquito innate immunity and provide a cellular link between local tissue cues and systemic immune outputs. Traditionally, mosquito hemocytes have been classified into granulocytes, oenocytoids, and prohemocytes. Granulocytes are phagocytic cells involved in immune surveillance and microbial clearance, whereas oenocytoids are commonly associated with melanization-related functions (43). Prohemocytes have generally been considered precursor-like populations with the potential to generate granulocyte and oenocytoid lineages. However, recent single-cell transcriptomic and flow-cytometry studies reveal substantially greater heterogeneity, including multiple granulocyte and oenocytoid subtypes, precursor-like populations, and infection-associated cellular states (39, 157–159).

Beyond their role as immune effector cells, hemocytes are dynamically shaped by physiological and infection-derived signals. Blood feeding alters hemocyte abundance, phagocytic activity, and patrolling behavior at the midgut basal lamina (40, 41, 93, 94, 126, 160). During *Plasmodium* infection, epithelial signals generated at the nitrated midgut basal lamina can recruit hemocytes and promote hemocyte-derived microvesicle release, thereby contributing to activation of complement-like immunity (48, 75). This response is also influenced by the resident microbiota, as prostaglandin release is reduced when mosquitoes infected with *P. berghei* are pretreated with antibiotics (75). These findings suggest that microbiota-dependent epithelial signals can help convert local midgut damage into systemic hemocyte activation. Consistent with this view, previous exposure to *Plasmodium* or bacteria can induce long-lasting increases in circulating granulocytes and enhance resistance to subsequent infection, and this primed state can be transferred through cell-free hemolymph from challenged mosquitoes (76). Importantly, these responses appear to depend in part on microbial exposure following parasite-induced epithelial disruption and involve hemocyte differentiation factor activity associated with Evokin/lipoxin signaling (161). Together, these studies place hemocyte priming within a tissue-level circuit linking epithelial damage, microbiota-derived signals, lipid mediators, and systemic immune readiness.

In addition to their role in systemic immune regulation, hemocytes can also influence arbovirus dissemination, although perturbation studies point to context-dependent effects. In *Ae. aegypti*, depletion of phagocytic hemocytes using clodronate liposomes reduced dissemination of Zika and dengue viruses to secondary sites, including salivary glands, legs, and ovaries (93), consistent with a role for phagocytic cells in promoting viral spread. In the same study, the authors demonstrated that phagocytic hemocytes become infected following dengue or Zika virus challenge and that transfer of the cellular fraction of mosquito hemolymph was sufficient to transmit infection to naïve mosquitoes (93), providing direct evidence for hemocyte-associated viral dissemination. The study also reported differential hemocyte recruitment to tissues relevant for horizontal and vertical transmission, including salivary glands and ovaries. By contrast, blockade of phagocytic capacity through latex bead injection has been reported to enhance systemic dissemination (94), suggesting that hemocyte phagocytosis can also contribute to containment of viral spread from the midgut. In this case, the authors observed minimal effects on midgut barrier integrity, suggesting that the increased dissemination was not primarily explained by changes in epithelial barrier disruption. Interestingly, latex bead injection also induced hemocyte recruitment to the midgut basal lamina (94). In this context, the increased dissemination observed after phagocytosis blockade could be interpreted in different ways. It may indicate that phagocytosis normally contributes to viral containment, or alternatively, that altered hemocyte behavior after bead injection indirectly affects dissemination, for example through changes in hemocyte recruitment and subsequent virus acquisition at the midgut interface. Furthermore, intrathoracic infection of phagocytosis-blocked mosquitoes resulted in higher viral loads, suggesting that hemocyte responses may influence viral replication or dissemination after systemic infection through mechanisms that remain unresolved (94).

The limited genetic tools in mosquito hemocyte biology have led to the development of alternative methodologies for immune cell manipulation. However, clodronate-mediated depletion and latex bead blockade also perturb hemocyte biology in different ways: the former reduces phagocytic hemocyte populations, whereas the latter interferes with phagocytic capacity and may also alter hemocyte activation, localization, or other cellular functions. Therefore, direct comparison of viral phenotypes resulting from these approaches requires consideration of the biological effects of each manipulation and the resolution of the viral readout. For example, quantification of viral RNA or titers in carcass samples after removal of the midgut provides evidence of virus beyond the initial midgut infection site but does not identify the specific secondary tissues contributing to this signal or distinguish among possible mechanisms underlying altered dissemination. these divergent outcomes should not be collapsed into a single pro- or antiviral role for hemocytes. Instead, they suggest that hemocytes perform multiple, separable functions during viral infection, where different perturbations selectively disrupt distinct functional axes, including containment through phagocytosis and potential contributions to systemic dissemination through hemocyte–virus interactions. Hemocytes may therefore influence viral outcomes in either direction depending on activation state, cellular subset, and tissue context (e.g. salivary glands, ovaries), rather than acting through a single unified mechanism.

These observations illustrate how hemocyte-dependent immune phenotypes can vary with exposure route, tissue interface, timing, and physiological state. Oral infection, septic injury, arboviral midgut infection, and *Plasmodium* traversal expose distinct tissues to different combinations of microbial products, epithelial damage, redox and metabolic stress, and blood-meal-induced signals. As a result, hemocytes may be recruited, activated, differentiated, or functionally engaged in different ways, producing outcomes that range from pathogen containment and complement-like killing to barrier maintenance, immune priming, or, in some contexts, viral dissemination. Hemocyte-dependent immunity is therefore best understood as part of a distributed tissue network that integrates physiological state with infection-derived cues, rather than as a uniform cellular defense program.

### 2.4 Endocrine signals tune immune allocation during reproductive physiology

#### 2.4.1 Hormonal and reproductive cues regulate immune architecture

Blood feeding and mating activate endocrine and metabolic programs that coordinate digestion, reproduction, and immunity. In anautogenous mosquitoes, the blood meal triggers amino-acid sensing, insulin/TOR signaling, and ecdysteroid production, which together support reproductive investment and egg development (35, 37, 162, 163). These pathways are not immune-specific, but they define the physiological framework in which immune responses occur. Ecdysone signaling can enhance antimicrobial and antiplasmodial responses (67, 164), and blood feeding itself can induce immune readiness against parasites or bacteria before pathogen-derived cues dominate (45, 165). These observations do not simply imply a single linear hormonal control of immunity. Instead, they suggest that endocrine state shapes immune potential by altering the physiological conditions under which pathogen exposure, tissue stress, and effector deployment occur. A central implication is that immune gene expression after blood feeding cannot be interpreted independently of reproductive physiology. Hormonal pathways such as ecdysone, juvenile hormone (JH), and insulin/TOR signaling can modulate complement-like factors, antimicrobial peptides, and epithelial programs, situating “immune activation” within a broader physiological transition that links metabolism, reproduction, tissue remodeling, and infection outcome. **Table 1** summarizes the major experimental findings demonstrating how endocrine state influences mosquito innate immunity and vector competence. Below, we discuss these findings in detail through the lens of three major hormonal pathways: juvenile hormone, insulin-insulin like growth factor, and ecdysone signaling.

#### 2.4.2 Juvenile hormone establishes the pre-blood-meal physiological and microbial baseline

Juvenile hormone (JH) is a major regulator of adult female mosquito physiology, particularly during the previtellogenic state. It coordinates adult maturation, metabolic readiness, tissue growth, and reproductive priming before blood feeding. In *Ae. aegypti*, JH signaling has been implicated in regulating midgut epithelial growth and shaping microbiota composition, in part through modulation of local immune activity (166). This baseline-setting role is not restricted to the midgut. In the fat body, JH signaling through its receptor Methoprene-tolerant (Met) and downstream transcriptional regulators such as Hairy and Groucho has been associated with broad changes in immune-gene expression (167, 168).

Consistent with a broad role for JH in immune and metabolic regulation, Met silencing alters the expression of immune genes, including antimicrobial peptides and components of the Toll and IMD pathways, alongside genes involved in energy metabolism and resource utilization (169). Whether these effects reflect direct transcriptional regulation of immune genes, indirect consequences of altered metabolic state, or crosstalk with ecdysone signaling remains unresolved. JH analog treatment has also been shown to increase Zika virus loads (170), further supporting a link between JH signaling and altered viral permissiveness. In addition, JH-dependent regulation of ribosomal and translational genes, which can support efficient viral replication, suggests another route through which endocrine state may influence infection success by altering the protein-synthetic capacity of mosquito tissues (171). Taken together, these findings suggest that the key question is not whether JH is simply immunosuppressive, but how it shapes the baseline physiological and metabolic landscape that determines how mosquitoes respond to subsequent blood feeding and infection.

#### 2.4.3 Insulin signaling links nutrition, hemocyte activity, and infection outcome

Insulin/insulin-like growth factor signaling (IIS) integrates blood-derived nutrition with reproductive physiology, metabolic state, and immune regulation. In mosquitoes, amino-acid availability after blood feeding regulates insulin-like peptide expression and downstream IIS activity through TOR-dependent signaling, thereby coordinating vitellogenesis, hemocyte activity, and infection-associated outcomes (42, 66, 148, 149, 172–175). IIS can also be engaged directly during infection through host- and parasite-derived signals, positioning this pathway as an important interface between metabolism and immunity.

In malaria infection, IIS can be engaged by vertebrate-derived cues, with detrimental consequences for mosquito physiology and parasite outcome. Mosquitoes feeding on malaria-infected vertebrate hosts can ingest elevated insulin levels, which alter mosquito Akt/ERK signaling and reduce their lifespan (176), indicating that IIS can be modulated by blood-borne host physiology. Consistent with this, elevated IIS activity has been associated with reduced antimicrobial peptide (AMP) expression, decreased survival after bacterial challenge and increased susceptibility to *Plasmodium* infections, suggesting an inverse relationship between IIS intensity and some immune outputs (147). In parallel, *Plasmodium* parasites can induce mosquito insulin-like peptides such as ILP3 and ILP4, which have been associated with immune suppression and increased parasite survival (177). The role of vertebrate-derived insulin in modulating mosquito immunity is further supported by findings from infection models beyond the *Anopheles*–*Plasmodium* interaction system. In *Ae. aegypti*, mosquitoes feeding on obese mice infected with Mayaro virus showed decreased infection and transmission rates, without significantly affecting viral replication within mosquitoes (178). Because obesity is associated with elevated circulating insulin levels, the investigators then tested whether insulin itself could account for this effect. Using Drosophila flies and cell culture models, the authors showed that insulin treatment reduced viral loads in an RNAi-independent manner by activating JAK/STAT signaling through ERK (179). Consistent with this mechanism, mosquitoes feeding on virally infected blood containing insulin showed reduced West Nile virus infection in *Culex* mosquitoes (179). In another study, treatment of *Ae. aegypti* with insulin decreased Chikungunya infection and transmission rates by modulating the expression of the Toll signaling members (180). Together, these findings highlight IIS as a signaling network integrating vertebrate- and pathogen-derived cues to shape mosquito immune and physiological outcomes.

Importantly, IIS is not a uniform systemic signal, but operates in a tissue- and time-dependent manner. Distinct insulin-like peptides, including ILP3, ILP4, and ILP5, show spatiotemporal expression patterns during infection and midgut invasion, indicating specialized roles rather than a single endocrine output (181). ILP3, in particular, links metabolism and reproduction by stimulating ecdysteroid production in the ovaries, further connecting IIS to endocrine coordination of reproductive investment (35). IIS also regulates hemocyte proliferation through insulin receptor signaling, thereby linking nutritional state to immune cell abundance and cellular immune capacity (42, 66). Despite these roles, sustained IIS pathway activation, such as Akt overexpression, can complicate this picture, as it can promote resistance to *Plasmodium* infection (173), a phenotype later associated with induced metabolic stress, altering reactive oxygen species (ROS) homeostasis, compromising midgut epithelial structure and reshaping immune responsiveness (174). These effects suggest that IIS does not simply enhance or suppress immunity. Rather, it tunes physiological trade-offs among metabolism, oxidative stress, reproduction, and immune capacity. Thus, IIS functions as a dose- and context-dependent regulatory hub that integrates nutritional signals, vertebrate- and parasite-derived cues, and tissue-specific responses. Its impact on vector competence depends not only on pathway activation itself, but on where, when, and in which physiological state that activation occurs, determining whether the outcome favors pathogen restriction, host tolerance, or increased permissiveness.

#### 2.4.4 Ecdysone signaling balances immune readiness with reproductive physiology

Ecdysone signaling provides a clear example of how a blood-meal-induced reproductive hormone can also reshape immune state. Following blood feeding, ecdysone levels rise and coordinate vitellogenesis, metabolism, tissue remodeling, and reproductive physiology (182). In *An. gambiae*, 20E has also been shown to prime immune responses that limit bacterial proliferation, control post-blood-meal microbiota expansion, and reduce *P. berghei* survival (67). In the same study, transcriptional analysis of a hemocyte-like cell line treated with 20E revealed regulation of multiple immune-associated genes, including antimicrobial peptides, CLIP-domain serine proteases, lysozymes, and prophenoloxidases (PPOs) (67). Consistent with a hemocyte-linked effector arm, 20E treatment induces expression of PPO3 and PPO8, two PPO family members enriched in the oenocytoid lineage, with PPO3 later shown to contribute to late-phase anti-*Plasmodium* immunity (158, 160). Pharmacological activation of ecdysone signaling using halofenozide similarly enhanced anti-*Plasmodium* responses, further supporting an immunomodulatory role of this pathway (67, 164). These studies support the idea that post-blood-meal endocrine signaling can increase selected immune capacities in *Anopheles*, particularly at the interface of microbiota control, hemocyte-associated effectors, and parasite survival.

The immunological effects of ecdysone, however, do not appear to be uniformly stimulatory across mosquito species or physiological contexts. In *Ae. aegypti*, ecdysone signaling has instead been linked to an immunity–reproduction trade-off, including reduced fat-body immune activity after blood feeding and bacterial challenge (37). In that study, activation of the ecdysone receptor (EcR) was proposed to suppress IMD signaling through regulation of the inhibitor Pirk, which interferes with amyloid signaling complexes formed between PGRP-LC and the IMD adaptor protein.

Differences between studies likely reflect variation in species, tissue context, timing, hormone concentration, and experimental design. Work in other insects is consistent with this interpretation, showing that 20E can produce concentration- and context-dependent phenotypic outcomes (183–185). Rather than acting as a simple immune activator or suppressor, ecdysone signaling appears to tune immune allocation according to reproductive and metabolic state. Under some conditions, transient 20E signaling may enhance selected immune effectors, microbiota control, or antiplasmodial activity; in others, sustained or tissue-specific signaling may constrain costly immune activation, particularly in compartments linked to reproductive investment. These findings argue against a binary view of endocrine regulation and suggest that vector competence may depend in part on when pathogen exposure occurs relative to the post-blood-meal endocrine program.

## 3 Discussion

### 3.1 A model of anticipatory and state-dependent mosquito immunity

Anticipatory immunity in mosquitoes is best understood as a property of physiological organization rather than as a discrete class of pathogen-specific responses. The central idea is not that mosquitoes “anticipate” infection in a pathogen-predictive manner. Instead, a blood meal is an intensive, organizing physiological event where concurrent cues—including endocrine shifts, altered metabolic states, tissue restructuring, and microbiota proliferation—collectively reshape mosquito biology to support reproductive output while simultaneously initiating remodeling of the gut, barrier tissues, and cellular immune compartments. The downstream effectors of these metabolic and reproductive cascades concurrently alter the innate immune architecture, establishing a state of transient immune preconditioning that bolsters defenses precisely during a window of high ecological risk. Importantly, time-resolved, midgut-specific transcriptomic studies demonstrate that this preconditioning lacks a long-term immunologic “memory”; if no pathogen is encountered, these elevated immune parameters gradually decay and return to baseline alongside the completion of the gonotrophic cycle (30, 31). This intricate nature of immune conditioning following a blood-meal does not replace the pathogen-specific responses, which may act independently of the blood-meal associated physiological alterations (**Table 1**). Rather, it reflects how immune preconditioning is dynamically interwoven with these changes, leading to transient physiological states that shape susceptibility before pathogen-derived signals dominate. Therefore, blood feeding is the key organizing event because it simultaneously enables pathogen acquisition and initiates systemic remodeling of the gut, microbiota, endocrine networks, barrier tissues, and cellular immune compartments.

**Figure 4:**
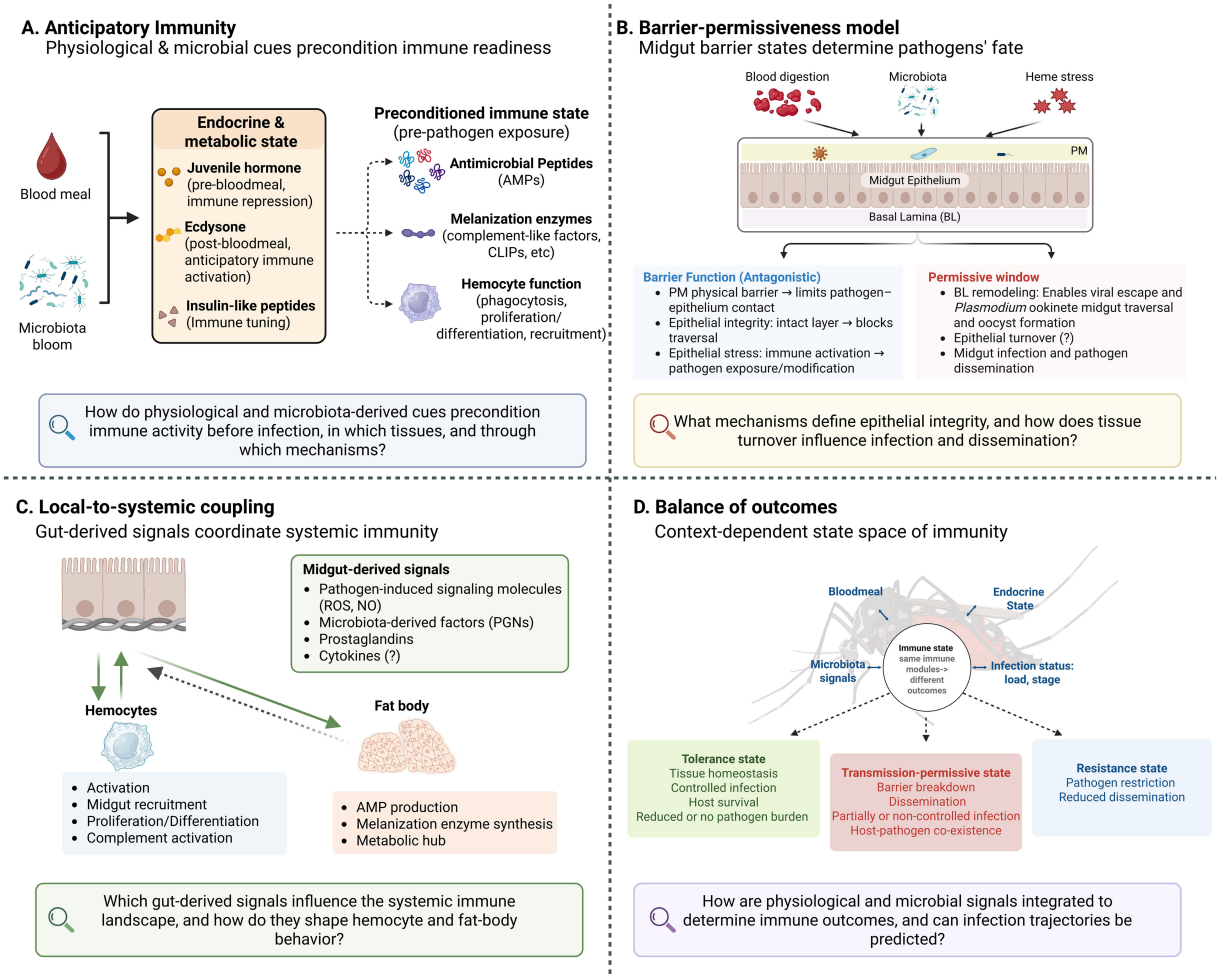
A state-dependent model linking blood feeding, microbiota, tissue barriers, and immune modules to vector competence. **(A)** Blood feeding and post-blood-meal microbiota expansion act as recurrent physiological cues that modulate endocrine, metabolic, epithelial, and immune processes, establishing a preconditioned immune state before pathogen-specific signals dominate. **(B)** Barrier-permissiveness model: the mosquito midgut functions as an integrative hub for cues derived from the blood meal, microbiota shifts, and heme-associated stress. These inputs promote epithelial repair, peritrophic matrix formation, basal lamina remodeling, and redox buffering, collectively shaping a tissue environment that can be more permissive or more restrictive for pathogen establishment. **(C)** Local–systemic coupling model: midgut-derived signals can promote hemocyte activation or redistribution and stimulate immune effector production by the fat body, linking local epithelial events to organism-wide immune state. **(D)** State-dependent immune outcome model: shared immune modules can produce different functional outcomes depending on physiological context, tissue interface, and pathogen stage. A given module may support barrier integrity or tissue repair in one context, promote pathogen restriction in another, or indirectly alter dissemination and transmission potential by changing tissue permissiveness. Created in BioRender. Samantsidis, G. (2026) https://BioRender.com/69bbng9.

This framework does not claim that blood feeding, microbiota, endocrine regulation, tissue barriers, or immune priming are newly recognized features of mosquito biology. Rather, its contribution is to treat these processes as coupled axes of a state-dependent immune system that determines how pathway activity is translated into resistance, repair, tolerance, or permissiveness (**Figure 4D**). Post-feeding endocrine and reproductive reprogramming, epithelial barrier dynamics, microbiota expansion, and gut-to-systemic signaling collectively generate immune states that are temporally structured and spatially compartmentalized. The endocrine axis, involving insulin-like signaling, juvenile hormone, and ecdysone, coordinates reproductive investment, metabolism, hemocyte behavior, and systemic responsiveness (**Figure 4A**). The barrier axis encompasses the physiological and structural consequences of blood digestion, including oxidative stress, peritrophic matrix formation, epithelial turnover, basal lamina remodeling, and tissue repair, which together shape whether pathogens establish in, traverse, or disseminate beyond the midgut (**Figure 4B**). Within this framework, microbiota-derived signals act as additional modulators of epithelial stress responses and immune gene expression, sometimes independently of direct pathogen recognition. Finally, gut-derived signals further propagate to systemic compartments, shaping hemocyte behavior and fat body function, thereby linking local infection status to systemic immune capacity (**Figure 4C**).

These axes operate on distinct time scales but converge at anatomical interfaces such as the midgut epithelium, basal lamina, hemocytes, fat body, and salivary glands. These interfaces correspond to the barriers through which vector competence is expressed, including midgut infection, midgut escape, systemic dissemination, and salivary gland invasion or escape. Vector competence therefore reflects how pathogen progression intersects with tissue state at each interface. Immune modules including Toll, IMD/Rel2, JAK/STAT, JNK, RNAi, and complement-like systems should not be viewed as isolated resistance pathways. Instead, they modify interface properties by shaping epithelial integrity, microbial context, effector availability, and hemocyte activity. Many apparent contradictions across studies may arise because experimental conditions shift which interface is limiting, or because different perturbations affect different layers of the same interface. Thus, divergent results can often be reinterpreted as different projections of a distributed causal system, while still leaving room for genuine methodological or biological variation that must be resolved directly.

From this perspective, mosquito immunity is best viewed as a dynamic tissue system shaped by ecological and physiological history. Anticipatory effects emerge when recurrent events such as blood feeding preconfigure pathogen-relevant interfaces before pathogen-specific recognition dominates. Immune function is therefore produced by the interaction of cue history, endocrine state, microbiota dynamics, epithelial and barrier architecture, and cellular immune activity. A key implication is that vector competence must be understood as a temporal process structured around the blood meal, which defines not only when pathogens enter the mosquito, but also the physiological sequence through which infection unfolds.

A central consequence of this framework is that vector competence, and by extension pathogen transmission, cannot be interpreted independently of the physiological state in which mosquito-pathogen interactions are measured. Increasing evidence from both experimental and field settings indicates that the efficacy of microbial and genetic control strategies can vary substantially across ecological contexts, consistent with the state-dependent nature of mosquito immunity. Studies have reported that symbiont-based approaches such as *Wolbachia* deployment can show heterogeneous performance across transmission settings (50). Of course, this does not imply that immune-state variation explains the variable performance of vector-control strategies in the field. Several *Wolbachia*-based interventions are supported by substantial experimental and field evidence (186–189), which has been comprehensively reviewed elsewhere (190). However, their performance can vary with mosquito genetic background, *Wolbachia* strain, pathogen genotype, environmental conditions, epidemiological baseline, and implementation context (191–194). Similarly, gene-drive systems may face additional challenges due to the evolution of resistance at targeted loci (195–197). Although these factors fall outside the scope of the mosquito state-dependent immune system framework developed here, it is plausible that mosquito microbial, physiological, and immune state represent one biological layer that interacts with established determinants of intervention performance, including mosquito genetic background, *Wolbachia* strain, pathogen genotype, implementation ecology, epidemiological baseline, climate, and targeted genetic loci modification (target-site resistance).

Finally, the context-dependence of mosquito vector competence becomes particularly relevant when considering environmental drivers such as temperature variation. Temperature fluctuations have been shown to reshape the transcriptomic landscape of blood-fed mosquitoes, with downstream effects on digestion, immune function, oogenesis, and oxidative stress management (198, 199). In the broader framework in which vector competence emerges from the integration of blood-feeding–associated physiological changes, microbiota dynamics, tissue barriers, and endocrine regulation, environmental temperature represents an additional axis of variation that can modulate mosquito immune state. Taken together, these observations suggest that variability in intervention outcomes is not solely attributable to technical or implementation factors but may also reflect unaccounted variation in mosquito physiological state, tissue organization, and ecological history. Therefore, a mechanistic understanding of mosquito immunity as a layered, state-dependent system is essential for improving the predictive power of vector competence assays and for designing interventions that will remain robust across environmental and epidemiological contexts.

### 3.2 Outstanding questions and experimental priorities in the field

This framework shifts the experimental problem from asking whether an immune gene or pathway affects infection to asking how that effect is produced within a defined physiological state. Pathway perturbations remain essential, but they need to be embedded in designs that separate the inducing cue, pathway activation, effector output, tissue state, pathogen stage, and transmission-relevant outcome. For mosquito immunity, this means tracing causal sequences that begin with ecological or physiological inputs, especially blood feeding, and proceed through endocrine, microbial, epithelial, and cellular immune changes before shaping pathogen establishment, traversal, dissemination, or transmission.

A first priority is time-resolved experimental design anchored around the blood meal. Because blood feeding rapidly reorganizes mosquito physiology, sampling should span multiple post-feeding windows and compare sugar-fed, non-infectious blood-fed, infectious blood-fed, pathogen-exposed, and physiologically manipulated states. This temporal structure is essential for distinguishing blood-meal-induced or preconditioned immune states from responses driven more directly by pathogen-derived cues. It is especially important because pathogen exposure often occurs within the same window as digestion, microbiota expansion, endocrine activation, and epithelial remodeling. Without this resolution, pathway activation measured after infection can easily be misinterpreted as pathogen-specific immunity when it may instead reflect feeding-induced systemic remodeling or an interaction between feeding state and pathogen stage.

A second priority is to treat microbiota and endocrine state as dynamic experimental variables rather than background conditions. Microbiota should be measured or controlled not only by presence or absence, but also by composition, load, spatial distribution, and activity, because blood feeding reshapes microbial ecology in ways that feed back onto epithelial stress, immune signaling, tissue repair, and vector competence. In parallel, endocrine pathways such as juvenile hormone, insulin-like signaling, and ecdysone should be manipulated and tracked as physiological regulators of immune allocation among reproduction, metabolism, barrier maintenance, and defense. Designs that combine microbial-state control with endocrine perturbation would be especially useful for separating direct pathway-mediated effects from indirect effects produced by altered host physiology. These systems jointly define the physiological context in which immune pathways operate.

A third priority is tissue-level resolution of immune function. Whole-organism and bulk transcriptional readouts remain valuable for discovery, but they cannot by themselves identify where a pathway acts or which tissue interface limits infection. Experiments should distinguish contributions from the midgut epithelium, basal lamina, hemocytes, fat body, and salivary glands, and should connect pathway activity to local changes in epithelial integrity, cellular behavior, barrier architecture, effector availability, and pathogen stage. This resolution is essential because vector competence is expressed through sequential interfaces, including midgut infection, midgut escape, systemic dissemination, and salivary gland invasion or escape, rather than through a single organism-wide immune output.

A final priority is to tie experimental outcomes to transmission-relevant stages rather than to single measurements of pathogen load. For arboviruses, this means distinguishing midgut infection, midgut escape, systemic dissemination, salivary gland infection, and transmission potential. For malaria parasites, equivalent stage-resolved measurements should distinguish parasite development in the blood bolus, ookinete traversal, oocyst establishment, sporozoite dissemination, salivary gland invasion, and transmission potential. The most informative experiments will combine cue manipulation, tissue-specific genetic perturbation, and stage-resolved pathogen measurement to determine whether a phenotype reflects resistance, tolerance, repair, altered permissiveness, or a shift in barrier integrity and function that limits transmission. In this way, mosquito immunity can be experimentally resolved as a dynamic physiological system shaped by blood-feeding history, microbiota, endocrine state, tissue architecture, and pathogen stage, rather than as a set of independent molecular pathways.
